# Applying Carbon Dots to Alleviate Photoinhibition and Boost Early Growth of Soybean Plants

**DOI:** 10.3390/plants15101446

**Published:** 2026-05-09

**Authors:** Marina M. Kawazoe, Adriana de Paula Cardoso, Marilza Castilho, Ailton J. Terezo, Adriano B. Siqueira, Halley C. Oliveira, Diego G. Gomes

**Affiliations:** 1 Department of Agronomy, State University of Londrina (UEL), Londrina 86057-970, PR, Brazil; marina.mologni@uel.br; 2 National Institute of Science and Technology in Nanotechnology for Sustainable Agriculture, INCT Nano Agro, Sorocaba 18087-180, SP, Brazil; marilza.terezo@ufmt.br (M.C.); ailton.terezo@ufmt.br (A.J.T.); adriano.siqueira@ufmt.br (A.B.S.); 3 Institute of Chemistry, Rede MT-Nano Agro, Federal University of Mato Grosso (UFMT), Cuiabá 78060-900, MT, Brazil; dridpaula@hotmail.com; 4 Laboratory of Biodiversity (LABIO), State University of Londrina (UEL), Londrina 86057-970, PR, Brazil

**Keywords:** carbon-based nanomaterials, environmental stresses, high-light stress, nanobiostimulants, nanotechnology

## Abstract

Although soybean is vital to the global economy, this crop faces productivity losses due to photoinhibition of photosystem II (PSII), which is worsened by heat and drought. Carbon dots (Cdots) offer a strategy to mitigate this stress by acting as light-harvesting and UV-protective agents. This study evaluated the foliar application of Cdots on soybean (*Glycine max* L. Merr. cv. BRS 1054 IPRO) exposed to high light intensity. In a greenhouse experiment with a completely randomized design, plants received deionized water (Control), synthesized Cdots at three concentrations (0.02, 0.05, and 0.20 mg mL^−1^), or a commercial Cdot product. Plants were grown under 50% shade and, at 24 days after sowing, transferred to a high-light greenhouse (20% attenuation). Measurements included PSII fluorescence (maximum quantum yield, potential activity, basal fluorescence, and dynamic photoinhibition) and leaf gas exchange (stomatal conductance, net photosynthesis, transpiration, intercellular CO_2_ concentration, intrinsic water use efficiency, and carboxylation efficiency), as well as chlorophyll index and growth traits. Cdots at 0.05 mg mL^−1^ and the commercial product maintained higher morning PSII maximum activity (+16% vs. Control), indicating enhanced photoprotection. Conversely, 0.20 mg mL^−1^ Cdots reduced PSII maximum activity by 62% at noon. At day 14, the 0.05 mg mL^−1^ treatment improved stress acclimation, reducing stomatal conductance and transpiration, while sustaining photosynthesis. Growth was significantly enhanced at this concentration, increasing chlorophyll content by 14%, shoot length by 26%, and total dry mass by up to 41% compared to controls. In conclusion, Cdots at 0.05 mg mL^−1^ alleviated chronic photoinhibition without increasing dynamic photoinhibition, thus acting as a promising nanobiostimulant that promotes soybean early growth under high-light stress.

## 1. Introduction

Climate change is exerting increasing pressure on natural and agricultural systems worldwide. Anthropogenic activities over the past century have raised atmospheric CO_2_ and other greenhouse gases, driving global warming and extreme weather events, such as heatwaves, droughts, intense precipitation, flooding, and altered freezing regimes [1]. These climate-driven stresses compromise plant physiological performance, increase susceptibility to diseases and pests, and, together, reduce growth, reproduction, yield, and survival, potentially threatening food security and societal stability [2].

Photosynthesis is particularly sensitive to these stresses. High irradiance combined with heat or water limitation creates an imbalance between light absorption and carbon fixation. In addition, stomatal closure and thermal stress limit CO_2_ assimilation, leading to excess excitation energy and the generation of reactive oxygen species (ROS), including superoxide radicals (O_2_^•−^), hydrogen peroxide (H_2_O_2_), hydroxyl radicals (^•^OH), and singlet oxygen (^1^O_2_). Accumulation of ROS damages photosystem II and disrupts its repair mechanisms [3,4,5,6].

To mitigate photodamage, plants regulate the coordination between photosystems I (PSI) and II (PSII). Photoprotective downregulation of PSII activity, characterized by reduced quantum efficiency and electron transport, together with increased non-photochemical quenching (NPQ), dissipates excess energy as heat and limits oxidative damage [7]. This reversible process, often referred to as dynamic photoinhibition, leads to a transient reduction in photosynthetic efficiency under excess light as a photoprotective mechanism. According to Tyystjärvi [8], it is important to distinguish dynamic photoinhibition from chronic photoinhibition; the former is mainly driven by rapid energy-dependent quenching (*qE*) in the antenna system and typically recovers within minutes to tens of minutes, whereas the latter involves degradation of the D1 protein and requires a more complex repair cycle that can take several hours. In this context, the apparent decrease in the maximum quantum yield of photosystem II (F_v_/F_m_) observed during short-term high-light exposure is often better interpreted as a regulated dissipation of excess energy rather than irreversible damage to PSII reaction centers.

In this context, a major challenge in modern agriculture is meeting the food demand of a growing global population, while at the same time mitigating climate change–induced yield losses and minimizing environmental impacts, highlighting the need for sustainable strategies to enhance crop adaptation [1]. Nanotechnology has emerged as a promising approach, offering precise and versatile solutions to improve crop resilience and productivity through targeted interactions at cellular and molecular levels. Applied via seed treatments, foliar sprays, or root uptake, nanomaterials can translocate through plant tissues, with their distribution governed by nanoparticle properties and plant species [9]. By enabling the controlled delivery of agrochemicals through biodegradable, lipid, silica, metallic, or carbon-based nanocarriers, nanotechnology enhances nutrient use efficiency, stress tolerance, and crop yield, while reducing chemical inputs, environmental impacts, and off-target toxicity, representing a sustainable alternative to conventional agricultural practices [10,11,12,13].

Among emerging nanotechnology-based strategies aimed at mitigating challenges in agrosystems and improving agricultural practices, carbon dots (Cdots) have gained attention as functional carbon-based nanomaterials with versatile physicochemical properties, including high diffusivity, tunable photoluminescence, rich surface chemistry, and biocompatibility with plant systems [14,15,16,17]. Through tailored synthesis strategies, Cdots exhibit controllable down- and up-conversion of light energy, enabling enhanced utilization of solar radiation by plant photosystems. Their interaction with chloroplasts has been associated with improved electron transport efficiency and stimulation of plant metabolic activity, resulting in increased photoassimilate production and photosynthetic performance. In addition, the ability of Cdots to be absorbed, translocated, and surface-functionalized supports their potential use as carriers of bioactive compounds with controlled or gradual release in plant systems [18,19,20].

Cdots derived from diverse sources have demonstrated positive physiological and agronomic effects across several crops, including rice, maize, pea, beans, soybean, wheat, lettuce, and tomato, depending on the mode of application [21]. Seed treatments have been associated with enhanced germination and early seedling vigor, through improved water uptake and modulation of aquaporin-related pathways. Foliar applications have consistently improved photosynthetic performance, biomass accumulation, and yield, while stress-targeted applications have increased tolerance to abiotic stresses, such as drought, salinity, and ultraviolet radiation, as well as biotic challenges involving bacterial and fungal pathogens. Additional emerging applications include post-harvest protection, where Cdots have shown antimicrobial activity in packaging systems. Overall, these applications demonstrate the potential of Cdots as a viable strategy to improve crop performance, stress resilience, and resource-use efficiency in sustainable agriculture.

In soybeans, Cdots exhibit multifunctional effects that depend on the mode of application and dosage. Soil (5 mg kg^−1^) and foliar applications (5 mg L^−1^) of Cdots synthesized from citric acid-based precursors enhance nitrogen availability, uptake, and utilization, particularly under drought conditions, by modulating root exudation, promoting beneficial microbiota and biological nitrogen fixation, and improving photosynthetic performance and metabolic activity. These responses lead to increased nitrogen accumulation and improvements in grain yield and protein content [22,23]. However, Cdots also display dose-dependent behavior, promoting growth and photosynthesis at low concentrations (100 mg L^−1^), while inducing phytotoxic effects at higher doses (≥500 mg L^−1^), highlighting the importance of optimized application rates for safe and effective agricultural use [24].

Soybean is a globally important crop with broad use in food, feed, and bioenergy systems. However, the production and trade of soybean are strongly affected by climate change [25], making this species a key crop model for assessing climate risks and resilience. Despite the promising optical properties of Cdots, including UV absorption and photoluminescence, their potential to mitigate photoinhibition and improve plant performance remains unexplored in soybean. Therefore, this study evaluated the effects of foliar application of Cdots synthesized from polyfunctional organic acids on early growth and photoprotection of soybean plants exposed to high-light conditions. We hypothesize that Cdots alleviate PSII chronic photoinhibition and enhance early growth of soybean plants under high-light stress.

## 2. Results

**Figure 1:**
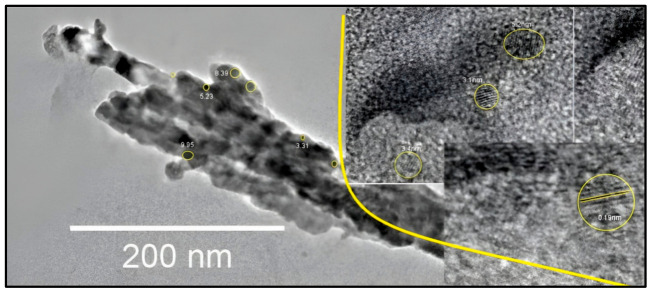
Transmission electron microscopy images of Cdots. Insets: The TEM image was zoomed in to show the interplanar distance of Cdots; the image was obtained with a magnitude of 20 nm.

The TEM analysis showed that Cdot particles could be observed with diameters of 3–10 nm (Figure 1). The interplanar distances in the highly crystalline carbon structure were approximately 0.19 nm, similar to interplanar spacings reported in previous studies [26].

**Figure 2:**
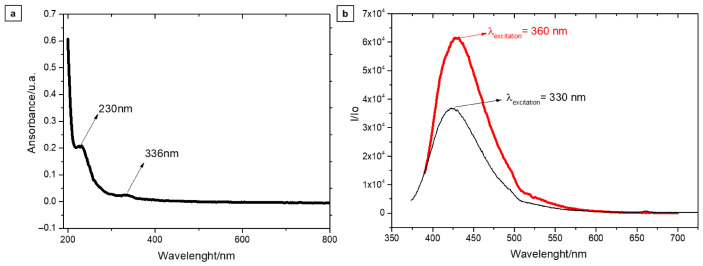
Spectroscopy analysis of Cdots. (**a**) UV-Vis spectra. (**b**) Photoluminescence spectra.

The UV–Vis spectrum exhibits two distinct absorption bands in the ultraviolet–visible region, both characteristic of luminescent nanocarbons (Figure 2a). The absorption band centered at 230 nm is attributed to π–π* electronic transitions within conjugated carbon systems, indicating the presence of a carbon core with mixed *sp*^2^/*sp*^3^ hybridization [26,27]. In addition, the absorption band with a maximum at 336 nm is associated with n–π transitions, which are characteristic of carbonyl functionalities, suggesting the presence of oxygen-containing functional groups on the surface of the Cdot samples [26,28].

Spectrofluorimetric analysis (PL) was carried out to investigate the fluorescence behavior of the Cdots and to evaluate the effect of excitation wavelength (λ_ex_) on their emission properties. Emission spectra were recorded using excitation wavelengths ranging from 280 to 550 nm to identify the conditions that maximize fluorescence intensity. The results show a consistent spectral evolution, with a maximum emission centered at approximately 430 nm (Figure 2b), indicating that the optimal excitation wavelength lies in the 330–360 nm range. This excitation-dependent emission behavior is attributed to multiple emissive surface states and heterogeneous energy levels associated with surface functional groups and structural defects, which enable selective population of different radiative recombination pathways upon variation in λ_ex_ [26,27,28,29]. The QY of the Cdots was 44.17%.

**Figure 3:**
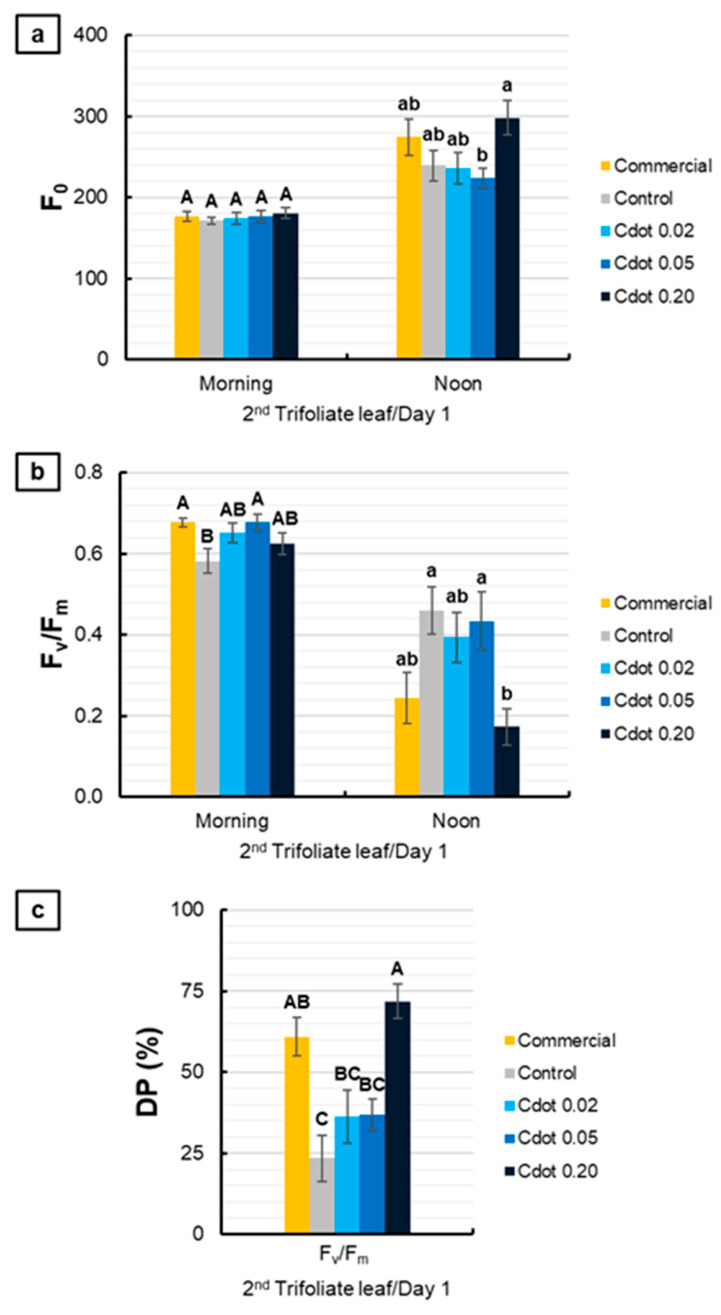
Basal fluorescence (F_0_) (**a**), maximum quantum yield of photosystem II (F_v_/F_m_) (**b**), and relative decrease in F_v_/F_m_ at noon (DP%) (**c**) of soybean plants (*Glycine max* L. Merr. cv. BRS 1054 IPRO), measured in the second trifoliate leaf on the first day after treatments with a carbon dot (Cdot) formulation at three different concentrations (0.02, 0.05, and 0.20 mg mL^−1^). The Commercial treatment represents a carbon dot-based product applied at the dose recommended by the manufacturer (0.20 mg mL^−1^). Plants treated with distilled water were used as the Control. Results are expressed as mean ± standard error (*n* = 8). Different uppercase letters above bars (morning) and different lowercase letters (noon) indicate significant differences among treatments at the same evaluation time, based on ANOVA followed by Tukey’s test (*p* < 0.05).

Figure 3 presents the evaluations performed on the second trifoliate leaf on the first day after the environmental change. For F_0_, no significant differences were observed among treatments in the morning. At noon, the Cdot treatments did not differ from the Commercial and Control treatments; however, Cdot 0.20 promoted a 33% increase in F_0_ compared with Cdot 0.05 (Figure 3a). Regarding F_v_/F_m_, in the morning, the Commercial and Cdot 0.05 treatments showed values 16% higher than the Control. At noon, only the Cdot 0.20 treatment differed from the Control, exhibiting a significant reduction of 62% (Figure 3b). Concerning DP (%), the Commercial and Cdot 0.20 treatments showed a significant increase compared with the Control. Notably, Cdot 0.20 exhibited a significant gain of 207% relative to the Control (Figure 3c).

**Figure 4:**
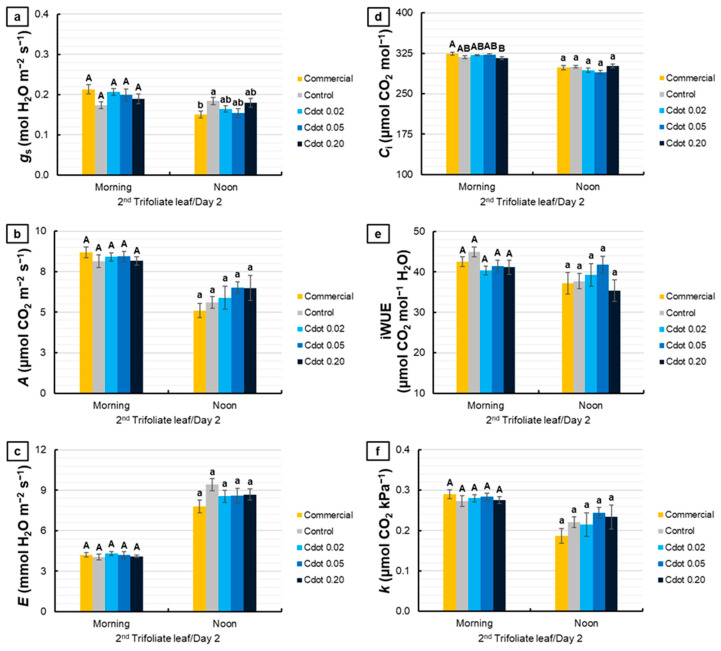
Stomatal conductance (*g*_s_) (**a**), net photosynthetic rate (*A*) (**b**), transpiration (*E*) (**c**), intercellular CO_2_ concentration (*C*_i_) (**d**), intrinsic water use efficiency (iWUE) (**e**), and instantaneous carboxylation efficiency (*k*) (**f**) of soybean plants (*Glycine max* L. Merr. cv. BRS 1054 IPRO), measured in the second trifoliate leaf on the second day after treatments with a carbon dot (Cdot) formulation at three different concentrations (0.02, 0.05, and 0.20 mg mL^−1^). The Commercial treatment represents a carbon dot-based product applied at the dose recommended by the manufacturer (0.20 mg mL^−1^). The Control was plants treated with distilled water. Results are expressed as mean ± standard error (*n* = 8). Different uppercase letters above bars (morning) and different lowercase letters (noon) indicate significant differences among treatments at the same evaluation time, based on ANOVA followed by Tukey’s test (*p* < 0.10).

Figure 4 shows the gas exchange evaluations performed on the second trifoliate leaf on the second day after the environmental change. For *g*_s_, no differences were observed among treatments in the morning. At noon, the Cdot treatments did not differ from the Control and Commercial treatments. However, the Commercial treatment promoted an 18% reduction in *g*_s_ compared with the Control (*p* < 0.10, marginally significant) (Figure 4a). *A* and *E* did not differ significantly among treatments in either evaluation period (Figure 4b,c). For *C*_i_, significant differences were observed only in the morning, with a 3% reduction in the Cdot 0.20 treatment compared with the Commercial treatment (*p* < 0.10, marginally significant) (Figure 4d). For iWUE and *k*, no significant differences were detected among treatments in either evaluation period (Figure 4e,f).

**Figure 5:**
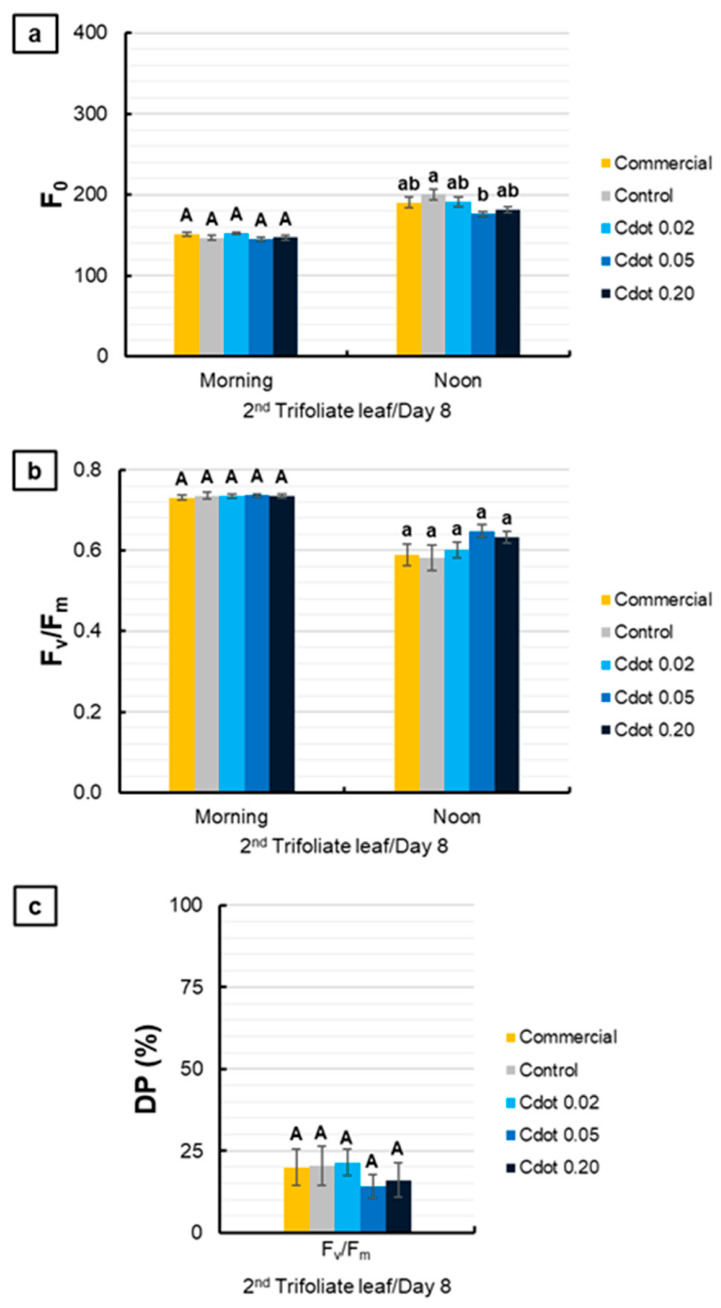
Basal fluorescence (F_0_) (**a**), maximum quantum yield of photosystem II (F_v_/F_m_) (**b**), and relative decrease in F_v_/F_m_ at noon (DP%) (**c**) of soybean plants (*Glycine max* L. Merr. cv. BRS 1054 IPRO), measured in the second trifoliate leaf on the eighth day after treatments with a carbon dot (Cdot) formulation at three different concentrations (0.02, 0.05, and 0.20 mg mL^−1^). The Commercial treatment represents a carbon dot-based product applied at the dose recommended by the manufacturer (0.20 mg mL^−1^). Plants treated with distilled water were used as the Control. Results are expressed as mean ± standard error (*n* = 8). Different uppercase letters above bars (morning) and different lowercase letters (noon) indicate significant differences among treatments at the same evaluation time, based on ANOVA followed by Tukey’s test (*p* < 0.05).

Figure 5 presents the evaluations performed on the second trifoliate leaf on the eighth day after the environmental change. For F_0_, no significant differences were observed among treatments in the morning. At noon, the Cdot 0.05 treatment reduced F_0_ by 12% compared with the Control (Figure 5a). For F_v_/F_m_ and DP (%), no significant differences were observed among treatments (Figure 5b,c).

**Figure 6:**
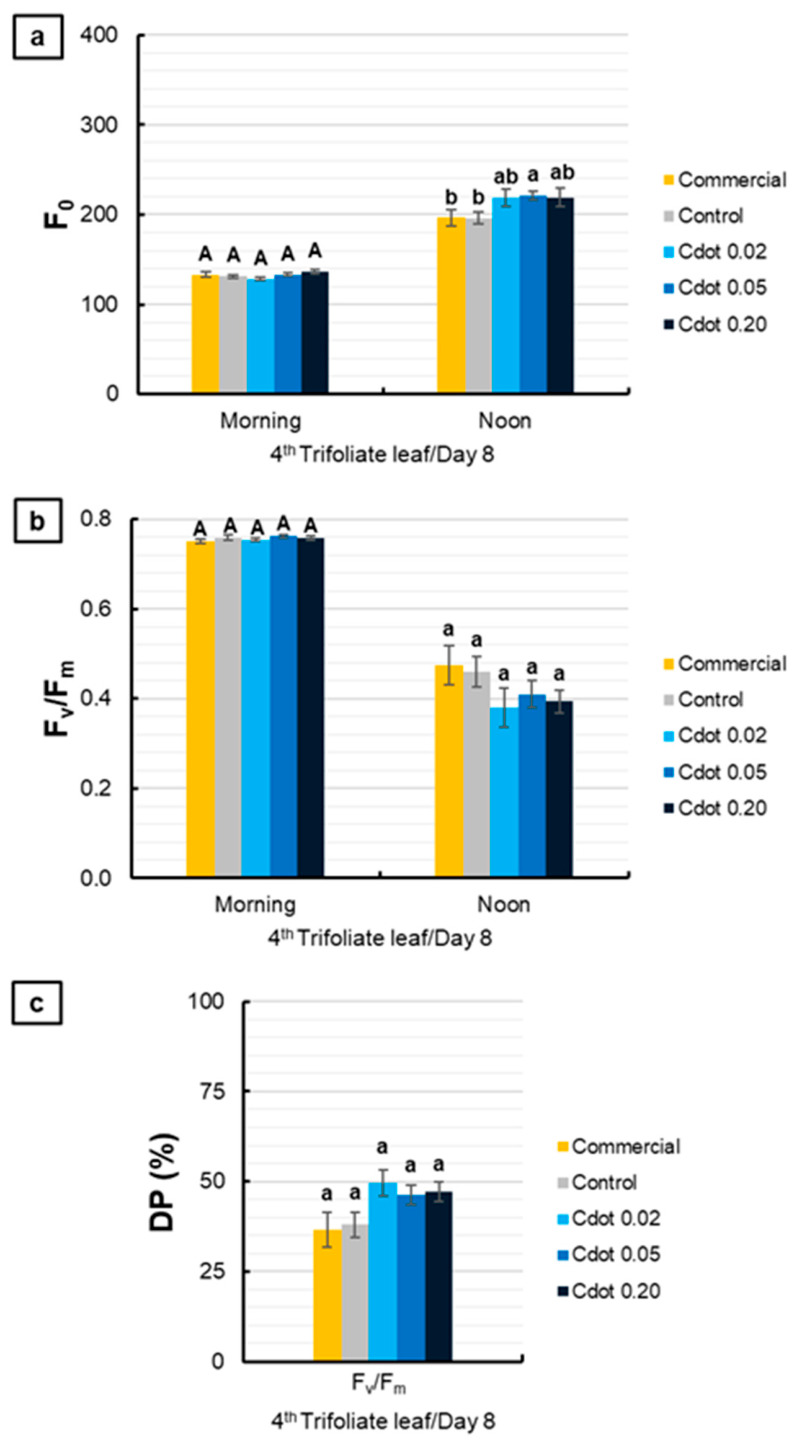
Basal fluorescence (F_0_) (**a**), maximum quantum yield of photosystem II (F_v_/F_m_) (**b**), and relative decrease in F_v_/F_m_ at noon (DP%) (**c**) of soybean plants (*Glycine max* L. Merr. cv. BRS 1054 IPRO), measured in the fourth trifoliate leaf on the eighth day after treatments with a carbon dot (Cdot) formulation at three different concentrations (0.02, 0.05, and 0.20 mg mL^−1^). The Commercial treatment represents a carbon dot-based product applied at the dose recommended by the manufacturer (0.20 mg mL^−1^). Plants treated with distilled water were used as the Control. Results are expressed as mean ± standard error (*n* = 8). Different uppercase letters above bars (morning) and different lowercase letters (noon) indicate significant differences among treatments at the same evaluation time, based on ANOVA followed by Tukey’s test (*p* < 0.10).

Figure 6 presents the results obtained from the fourth trifoliate leaf on the eighth day after the environmental change. For F_0_, no significant differences were observed among treatments in the morning. At noon, the Cdot 0.05 treatment increased F_0_ by approximately 13% compared with the Commercial and Control treatments (*p* < 0.10, marginally significant) (Figure 6a). For F_v_/F_m_ and DP (%), no significant differences were observed among treatments (Figure 6b,c).

**Figure 7:**
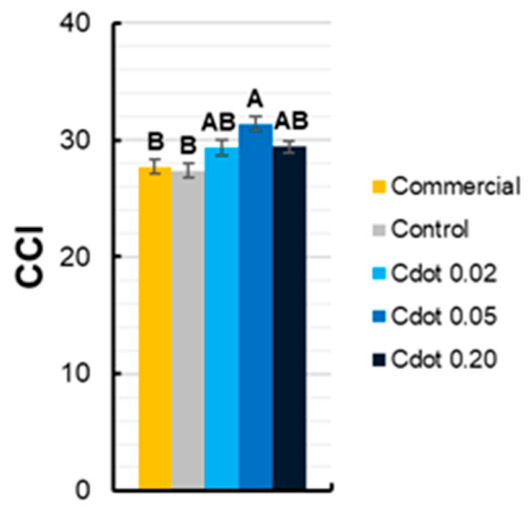
Chlorophyll content index (CCI) of soybean plants (*Glycine max* L. Merr. cv. BRS 1054 IPRO) measured in the fourth trifoliate leaf at 36 days after sowing. The treatments were carbon dot (Cdot) formulations at three different concentrations (0.02, 0.05, and 0.20 mg mL^−1^). The Commercial treatment represents a carbon dot-based product applied at the dose recommended by the manufacturer (0.20 mg mL^−1^). Plants treated with distilled water were used as the Control. Results are expressed as mean ± standard error (*n* = 8). Different uppercase letters above bars indicate significant differences among treatments within the same trifoliate leaf and evaluation day, based on ANOVA followed by Tukey’s test (*p* < 0.05).

Figure 7 demonstrates the CCI values. The Cdot 0.05 treatment resulted in a significant 14% increase in CCI compared with the Commercial and Control treatments.

**Figure 8:**
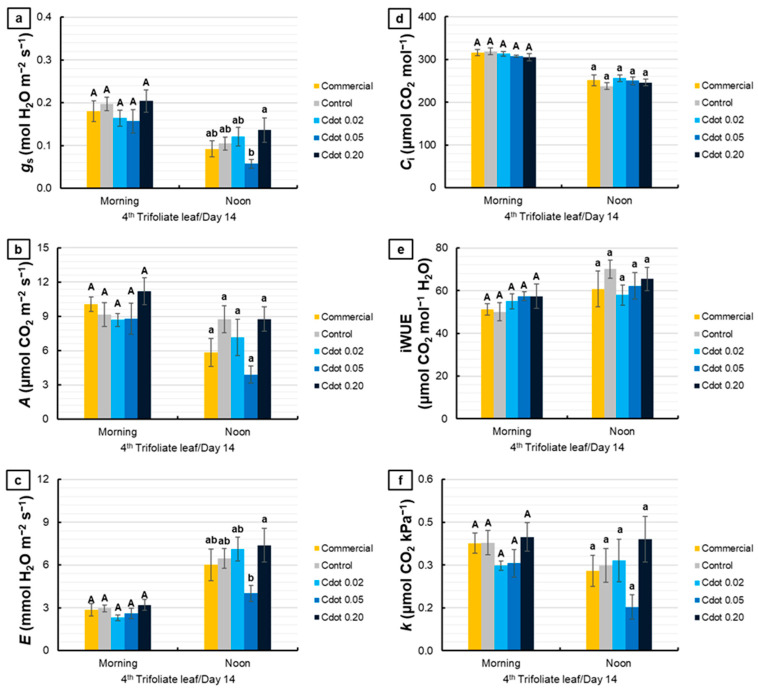
Stomatal conductance (*g*_s_) (**a**), net photosynthetic rate (*A*) (**b**), transpiration (*E*) (**c**), intercellular CO_2_ concentration (*C*_i_) (**d**), intrinsic water use efficiency (iWUE) (**e**), and instantaneous carboxylation efficiency (*k*) (**f**) of soybean plants (*Glycine max* L. Merr. cv. BRS 1054 IPRO), measured in the fourth trifoliate leaf on the 14th day after the treatments with a carbon dot (Cdot) formulation at three different concentrations (0.02, 0.05, and 0.20 mg mL^−1^). The Commercial treatment represents a carbon dot-based product applied at the dose recommended by the manufacturer (0.20 mg mL^−1^). Plants treated with distilled water were used as the Control. Results are expressed as mean ± standard error (*n* = 6). Different uppercase letters above bars (morning) and different lowercase letters (noon) indicate significant differences among treatments at the same evaluation time, based on ANOVA followed by Tukey’s test (*p* < 0.10).

Figure 8 presents the gas exchange evaluations performed on the fourth trifoliate leaf on the 14th day after the environmental change. For *g*_s_, no significant differences were observed among treatments in the morning. At noon, the Cdot treatments did not differ from the Control and Commercial treatments. However, the Cdot 0.05 treatment showed a 58% reduction in *g*_s_ compared with Cdot 0.20 (*p* < 0.10, marginally significant) (Figure 8a). For *A*, no significant differences were observed among treatments in either evaluation period (Figure 8b). For *E*, no differences were detected in the morning. At noon, the Cdot 0.05 treatment exhibited a 46% reduction in *E* compared with Cdot 0.20 (*p* < 0.10, marginally significant), while the remaining treatments did not differ from each other (Figure 8c). For *C*_i_, iWUE, and *k*, no significant differences were observed among treatments in either evaluation period (Figure 8d–f).

**Figure 9:**
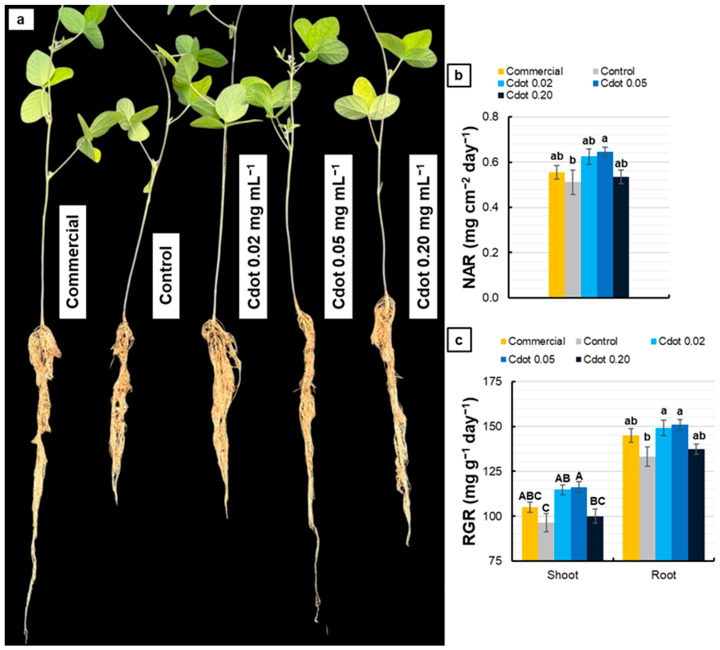
Representative photograph (**a**), net assimilation rate (NAR) (**b**), and relative growth rate (RGR) (**c**) of soybean plants (*Glycine max* L. Merr. cv. BRS 1054 IPRO) treated with a carbon dot (Cdot) formulation at three different concentrations (0.02, 0.05, and 0.20 mg mL^−1^). The Commercial treatment represents a carbon dot-based product applied at the dose recommended by the manufacturer (0.20 mg mL^−1^). The Control group is plants treated with distilled water. Results are expressed as mean ± standard error (*n* = 8). For RGR, different uppercase letters above bars (shoot) and different lowercase letters (root) indicate significant differences among treatments within the same plant organ, based on ANOVA followed by Tukey’s test (*p* < 0.05).

Figure 9 shows a representative photograph of plants subjected to different treatments at 38 DAS (Figure 9a), along with the NAR and RGR. The Cdot 0.05 treatment promoted a 27% increase in NAR compared with the Control (Figure 9b). Both shoot and root RGR values showed significant differences only for the Cdot 0.02 and Cdot 0.05 treatments compared with the Control, with average increases of 20% in shoots and 13% in roots (Figure 9c).

At 38 DAS, as shown in Table 1, the Cdot 0.05 treatment resulted in an average 25% increase in SL compared with the Commercial, Control, and Cdot 0.20 treatments. For RL, no differences were observed among treatments. Regarding LA, the Cdot 0.20 treatment showed an 18% reduction compared with Cdot 0.05. For LDM, SDM, and RDM, the Cdot treatments did not differ from the Commercial treatment. Compared with the Control, the Cdot 0.05 treatment promoted increases of 24% in LDM (*p* < 0.10, marginally significant) and 26% in RDM. Additionally, the Cdot 0.02 and Cdot 0.05 treatments promoted, on average, increases of 41% in SDM and 34% in TDM compared with Control.

## 3. Discussion

The TEM analysis of Cdots is related to the limited stability of these nanoparticles and their pronounced tendency to agglomerate, as illustrated in Figure 1. Although agglomerates were observed in multiple regions of the support grid across all analyzed samples, identifying non-aggregated regions enabled the acquisition of high-quality images of individual Cdot nanoparticles, allowing assessment of their size, morphology, and interplanar spacing. Cdot particles could be observed with a diameter of 3–10 nm. The interplanar distances in the highly crystalline carbon structure were approximately 0.19 nm, similar to interplanar spacings reported in previous studies [26]. Interplanar spacings of approximately 0.21, 0.204, and 0.20 nm were observed, which are consistent with the (100) and (102) crystallographic planes of graphitic sp^2^-hybridized carbon [26,27]. The morphological properties indicate that the Cdot was formed via light-induced quantum confinement.

The bands observed in the UV-Vis region confirmed the formation of the inner region, composed of carbon atoms, and the outermost region of the Cdots, composed of functional groups, mainly the carbonyl group. The UV-Vis absorption study was used to evaluate the emission behavior of the Cdots. Excitation at 360 nm showed twice the emission intensity of excitation at 330 nm. The results show that UV light is absorbed by the Cdots, with the respective emission at visible light, at 430 nm. Two points can be emphasized. Firstly, we observe that Cdots exhibit high UV absorption, specifically at wavelengths below 230 nm. This indicates that excessive UV light, which is harmful to plant development, can be attenuated by using Cdots [30].

In this study, we investigated the dual role of Cdots in plant growth and photoprotection by establishing a dose range informed by preliminary assays. A broad screening of materials and concentrations identified 0.05 mg mL^−1^ as the optimal dose for biological activity. Based on this, we evaluated a gradient from 0.02 to 0.20 mg mL^−1^, including the optimal dose, a concentration equivalent to the commercial product, and a tenfold dilution of the commercial dose. We further assessed whether Cdots could not only stimulate soybean early growth but also enhance tolerance to light stress. Particular emphasis was placed on the transition to high irradiance, a critical phase in which dynamic photoinhibition often impairs the photosynthetic apparatus. In a study with maize, Gomes et al. [31] demonstrated that a sudden shift from a shaded environment to high irradiance induces pronounced dynamic photoinhibition during the first days of exposure, particularly in leaves developed under low light, validating this experimental system as a robust tool for assessing the functional stability of PSII during light acclimation. To characterize these responses, chlorophyll a fluorescence parameters, gas exchange, and biometric traits were measured throughout the acclimation of the plants to the new light environment.

In general, on the first day after the environmental change, chlorophyll a fluorescence parameters indicated rapid and sensitive responses of PSII (Figure 3). Measurements taken in the morning, before the onset of peak light intensity, revealed that the Commercial and Cdot 0.05 treatments had F_v_/F_m_ values about 16% higher than the Control (Figure 3b), suggesting an alleviation of chronic photoinhibition. This improved baseline photochemical performance likely contributed to the more efficient acclimation observed in these treatments. However, unlike the Cdot 0.20 and Commercial treatments, Cdot 0.05 did not exhibit exacerbated dynamic photoinhibition (DP%) at noon (Figure 3c). Together, these parameters indicate a greater functional stability of PSII during the initial acclimation phase in the Cdot 0.05 treatment.

The reduction in the F_v_/F_m_ ratio may result from either a decrease in PSII photochemical efficiency or an increase in non-photochemical energy dissipation. Limitations in PSII photochemistry promote an increase in F_0_ due to impaired oxidation of quinone A (*Q*_A_) and the consequent restriction of electron flow, indicating disturbances in the electron transport chain and a decline in PSII photochemical activity, whereas enhanced non-photochemical dissipation leads to a concomitant reduction in F_0_ and F_m_ [32,33,34]. This behavior was clearly described by Gomes et al. [31], who observed a significant increase in F_0_ and a decrease in F_v_/F_m_ during the first days of exposure of maize plants to high irradiance, which was interpreted as a temporary limitation of *Q*_A_ oxidation and intensified excitatory pressure on the PSII reaction centers. In this context, on the first day of evaluation, the increase in F_0_ observed in the Cdot 0.20 treatment, together with the marked reduction in F_v_/F_m_, especially at noon, indicates structural and functional disturbances of the PSII reaction centers, reflecting acute photochemical stress under high irradiance.

Although Cdots are generally described as nanomaterials with low toxicity and high biocompatibility [21,35], some uncertainties remain. Previous studies have shown that Cdots can be absorbed and translocated within plant tissues, with the potential to accumulate in different organs and even reach the cell nucleus [36,37]. In addition, their interaction with soil, including effects on microbial communities and enzymatic activity, suggests that indirect ecological impacts cannot be excluded, particularly given the limited understanding of their long-term environmental fate [21,37]. Plant responses are strongly concentration-dependent, with low concentrations promoting growth and higher concentrations potentially reducing biomass and inducing oxidative stress [35,37,38]. In this study, the concentration of 0.20 mg mL^−1^ did not cause visible phytotoxicity or persistent impairment in growth and physiological performance, being associated only with transient photochemical stress. This is consistent with Serafim et al. [26], who reported no toxicity in zebrafish embryos and no phytotoxicity in soybean and maize at comparable concentrations using Bio-Cdots derived from swine effluent, materials with diameters smaller than 10 nm, a crystalline graphitic structure, and photoluminescence in the visible range. Although obtained through a different synthesis route, these materials share similar physicochemical characteristics with the Cdots used in this study. Taken together, these findings provide converging evidence that Cdots with comparable structural and optical properties, regardless of whether they are derived from chemical precursors or complex biowaste, are unlikely to cause detrimental effects within the concentration ranges typically used for plant growth promotion. Nevertheless, further studies addressing chronic exposure, accumulation dynamics, and soil–plant interactions are still needed to better define their safety limits in agricultural systems.

Corroborating this, Chen et al. [39] demonstrated that Cdots synthesized from citric acid and urea, exhibiting broad spectral absorption (300–700 nm) and low fluorescence quantum efficiency (2.11%), modulate light energy distribution in Chlorella pyrenoidosa under high irradiance. Foliar application of Cdots reduced excessive light absorption, with optimal effects observed at the concentration of 0.04 mg mL^−1^, attenuating the incident energy load without compromising the light required for photosynthesis. Fluorescence analyses indicated that high-light stress increased the energy absorbed and dissipated by the reaction centers, reflecting PSII saturation and heightened excitation pressure. In contrast, the presence of Cdots promoted adjustments in energy partitioning, alleviating the overload of the reaction centers and enhancing the photochemical utilization of absorbed energy, with reduced reliance on regulated thermal dissipation.

These patterns are consistent with those reported by Gomes et al. [31] in maize plants subjected to abrupt changes in light conditions. Although soybean (C3) and maize (C4) differ in the metabolic organization of carbon assimilation, the primary photochemical processes underlying dynamic photoinhibition of PSII are highly conserved between C3 and C4 species. Accordingly, the similarity of the initial responses, characterized by a transient increase in F_0_ and a decrease in F_v_/F_m_, reflects universal mechanisms of photochemical limitation and increased excitation pressure during acclimation to high irradiance. However, differences in carbon fixation pathways and photosynthetic energy balance modulate the extent and reversibility of photoinhibition. C4 species maintain the CO_2_ supply to Rubisco through a carbon-concentrating mechanism, which reduces excitation pressure and limits chronic photoinhibition under high-light and high-temperature conditions. In contrast, C3 species rely more heavily on NPQ for photoprotection and are therefore more prone to dynamic photoinhibition under drought or heat, with repeated or prolonged stress potentially leading to irreversible photodamage [33].

Li et al. [40] evaluated the protective effect of a Cdot formulation synthesized from citric acid via a microwave-assisted method, applied to rice cell suspension cultures under various abiotic stresses. The authors demonstrated that application of Cdots at 0.1 mg mL^−1^ completely mitigated dynamic photoinhibition in rice cells, promoting biomass accumulation under light stress. According to the authors, the protective effect of Cdots is linked to their ability to reduce ROS accumulation under stress, as well as to enhance the activity of antioxidant enzymes, levels of phenolic compounds and flavonoids, and nutrient assimilation.

In line with these findings, Lei et al. [41], working with foliar application of ^13^C-labeled Cdots, showed that these nanomaterials can be absorbed by leaves and subsequently translocated within the plant. Their results indicate that Cdots are incorporated into central metabolic pathways, contributing to the formation of compounds such as glucose, sucrose, and organic acids. This suggests that, in addition to modulating ROS balance and antioxidant responses, Cdots may also influence plant performance through direct metabolic contributions.

Despite pronounced differences in photochemical parameters, gas exchange measurements on the second trifoliate leaf remained stable across treatments and evaluation periods (Figure 4). The absence of significant variations in *A*, *E*, *C*_i_, iWUE, and *k* indicates that Cdots did not impose stomatal or biochemical limitations on carbon assimilation. A consistent partial stomatal closure, reduction in *A*, and increase in *E* were observed at noon across all treatments when comparing evaluation periods, an effect attributed to the natural noon depression of photosynthesis [42]. These results suggest that the intensification of dynamic photoinhibition associated with the higher Cdot dose (0.20 mg mL^−1^) was transient and did not persist after the plants were transferred to the new environment.

As acclimation progressed, eight days after the environmental change, the beneficial effects of the Cdot 0.05 treatment became more pronounced. On the eighth day, the reductions in F_0_ observed in the second trifoliate leaf, developed under lower irradiance conditions, indicate lower damage in PSII reaction centers (Figure 5). In contrast, in the fourth trifoliate leaf, which expanded under high-light conditions, particularly at noon, a distinct pattern was observed: Cdot 0.05 promoted an increase in F_0_ without concurrent reductions in F_v_/F_m_ compared to the Control (Figure 6).

This behavior suggests that, in leaves developed under high irradiance, the increase in F_0_ may reflect a physiological adjustment to the higher light load, without compromising PSII photochemical efficiency, representing a process of structural and functional acclimation to the new light environment. Similar results were reported by Gomes et al. [31], who observed greater sensitivity to dynamic photoinhibition in maize leaves formed under low irradiance, whereas leaves developed after transfer to high light exhibited greater F_v_/F_m_ stability, indicating progressive acclimation of the photosynthetic apparatus. In the Cdot 0.05 treatment, a concomitant increase in the chlorophyll content index was observed (Figure 7), suggesting a higher concentration of photosynthetic pigments.

According to Baker [43], increases in pigment content can lead to elevated F_0,_ due to a higher leaf light absorption coefficient, as well as to optical and structural changes associated with the organization of antenna complexes and connectivity between PSII reaction centers. Under these conditions, the rise in F_0_ is not necessarily indicative of damage or inactivation of reaction centers, but may instead reflect structural adjustments of the photosynthetic apparatus during the acclimation of new leaves, provided that photochemical efficiency parameters, such as the F_v_/F_m_ ratio, remain stable. Supporting this interpretation, the stability of F_v_/F_m_ across treatments indicates that the maximum quantum efficiency of PSII was maintained, showing that the treatment with Cdots did not contribute to permanent intensification of stress and was insufficient to cause chronic inhibition of photosynthesis.

In agreement with the previous data, quantification of DP (%) over the measurement days reinforces the occurrence of acute photochemical stress, induced by the environmental change and enhanced by treatment with Cdot 0.20 and the commercial product on the first day of acclimation (Figure 3c). This temporal pattern is consistent with observations by Gomes et al. [31], who reported high energy dissipation values during the first days after exposure to high irradiance, followed by a progressive reduction in DP (%) as plants acclimated to the new light regime. Although such responses are essential for photoprotection, their initial magnitude indicates a transient imbalance between energy absorption and utilization, with subsequent re-establishment of the energy balance and recovery of PSII photochemical efficiency.

Fourteen days after the environmental change, in the trifoliate leaf developed under high irradiance the reduction in *g*_s_ in plants treated with Cdot 0.05 was not accompanied by a decline in *A* (Figure 8). This behavior suggests more efficient coordination between stomatal regulation and carbon assimilation. Maintenance of assimilation despite lower stomatal conductance may be associated with increased chlorophyll concentration, enhancing the capture and utilization of light energy and promoting greater photosynthetic efficiency at the chloroplast level. Collectively, these responses reflect a more efficient acclimation process and may contribute to sustained growth and biomass accumulation under high irradiance.

The physiological advantages conferred by the Cdot 0.05 treatment were clearly reflected in growth responses. The increase in NAR indicates greater carbon gain per unit of leaf area, consistent with enhanced photochemical performance and higher chlorophyll content. Additionally, the increase in RGR values of both the shoots and roots demonstrates that the stimulatory effects of Cdots at concentrations of 0.02 and 0.05 mg mL^−1^ were systemic and not limited to shoot tissues, possibly linked to improved nutrient assimilation and greater metabolic efficiency, supported by enhanced photosynthetic performance (Figure 9).

It is noteworthy that *A* measured using a portable infrared gas analyzer (IRGA) did not differ significantly among treatments at the evaluated time points. However, the Cdot 0.05 treatment showed the highest leaf area, which may result in a higher CO_2_ assimilation per plant. Even though, such instantaneous measurements may not fully capture daily carbon assimilation, particularly under high and fluctuating irradiance, where photochemical efficiency and recovery dynamics vary throughout the day. In contrast, higher NAR values observed in Cdot 0.05 plants (Figure 9b) integrate carbon gain over time at the whole-plant level and therefore provide a more relevant link to biomass accumulation than instantaneous leaf-level A measurements. Together with the increased chlorophyll content (Figure 7) and improved PSII functional stability (Figure 5), these results support the interpretation that Cdot 0.05 enhanced photosynthetic performance, contributing to the observed increases in biomass.

Overall, these findings are consistent with the interpretation that Cdot 0.05 exerted both photoprotective and biostimulant effects, leading to improved photosynthetic performance and growth. The photoprotective component is supported by greater PSII stability without increasing dynamic photoinhibition under high irradiance, while the biostimulant component is reflected in enhanced chlorophyll content, improved integrated carbon gain over time, and increased biomass accumulation. The biomass differences are not necessarily explained by instantaneous increases in *A* alone and may also involve enhanced light use efficiency, alleviation of chronic photoinhibition, and altered carbon allocation patterns, as reported in previous studies [39,40].

Supporting these findings, Cdots have been shown, through different application methods, to modify nitrogen bioavailability and promote soybean growth even under stress conditions. Wang et al. [22] demonstrated that soil application of Cdots (5 mg kg^−1^), synthesized from citric acid and ethylenediamine, increased nitrogen availability in the soil, enhanced biological nitrogen fixation in nodules, and modulated root exudates, favoring the recruitment of beneficial microbiota and the uptake of nitrogen and water under drought. Similarly, Ji et al. [23] reported that foliar application of Cdots (5 mg mL^−1^) reduced ROS accumulation in soybean leaves under water stress, stimulated photosynthetic activity and carbohydrate translocation, and increased the synthesis of beneficial metabolites, while regulating root exudates. These effects led to higher nitrogen levels in roots and shoots, resulting in increased crop yield and grain protein content.

The growth and biomass parameters support this interpretation (Table 1). Increases in shoot length, leaf dry mass, and root dry mass under the Cdot 0.05 treatment suggest more efficient carbon allocation and balanced vegetative development, consistent with the maintenance of PSII photochemical efficiency and more effective use of absorbed energy, as indicated by chlorophyll fluorescence parameters. In contrast, the reduction in leaf area observed in plants treated with Cdot 0.20 indicates that higher doses negatively affect leaf expansion, likely as a consequence of initial photochemical stress.

Similar results were reported by Serafim et al. [26], who evaluated the foliar application of Cdots synthesized from swine effluents on soybean plants and observed increases in root growth, plant height, leaf area, and stem dry mass, particularly at lower doses (0.010 and 0.033 mg mL^−1^). These findings further highlight the potential of Cdots as growth-promoting agents, with effects likely associated with multiple mechanisms. They can act multifunctionally, modulating light absorption and redistribution, attenuating ROS accumulation, and stimulating both antioxidant defense systems and metabolic processes involved in carbon and nutrient assimilation. Through these actions, Cdots enhance the physiological resilience of plants to environmental stresses, positioning this nanotechnology as a high-potential tool for sustainable agricultural applications and for use in scenarios of increasing climate instability [21,35].

## 4. Materials and Methods

### 4.1. Synthesis and Characterization of Carbon Dots

The synthesis of Cdot formulation was carried out from polyfunctional organic acids, using a methodology adapted from the literature [44].

The absorption spectra of the samples were measured using a Lambda 265 UV-Vis spectrophotometer (PerkinElmer, Waltham, MA, USA) with a xenon-pulsed source. Measurements were taken at room temperature and the scan comprised excitation wavelengths from 200 to 800 nm. The samples were arranged in quartz cuvettes, with a 10 mm optical path for analysis.

The emission spectra of the samples were measured using an ISS PC1 photon-counting spectrofluorometer (ISS, Inc., Champaign, IL, USA), operating with a 300-watt xenon lamp powered by a stabilized current of 15 amperes. The excitation and emission monochromators for sample measurements used a spectral slit width of 1.0 mm. The excitation wavelength was increased by 20 nm, spanning the range 280 to 500 nm, and the emission was recorded from 300 to 700 nm. The samples for emission analysis were arranged in quartz cuvettes with a 10 mm optical path.

Determination of the quantum yield (QY) of the samples was performed using the values of the integrated photoluminescence intensity and the absorbance of the samples and related to standard quinine sulfate, as indicated in the literature. An acidic solution (0.1 mol L^−1^ H_2_SO_4_) of quinine sulfate at 1 × 10^−5^ mol L^−1^ was prepared from quinine sulfate dihydrate reagent from Fluka, with a molecular mass equal to 782.96 g mol^−1^ [27,28].

Transmission Electron Microscopy (TEM) images of the samples were obtained using a FEI^®^ Tecnai G2-20 SuperTwin 60–200 kV (FEI Company, Hillsboro, OR, USA), with a: nominal calibrated magnification range in TEM mode from 340× to 1,050,000×, spherical aberration coefficient of Cs = 2.0 mm, line resolution of 0.24 nm and point resolution of 0.1 nm, maximum stage tilt angle of ± 45° on the α axis and ± 30° on the β axis, and maximum stage tilt angle of ±65° on the α axis with the tomography sample holder.

### 4.2. Plant Material and Experimental Design

Soybean (*Glycine max* L. Merr.) seeds from the cultivar BRS 1054 IPRO were supplied by the Brazilian Agricultural Research Corporation (EMBRAPA–Soybean, Londrina, Brazil). The experiment was conducted in a completely randomized design in two greenhouses belonging to the Center for Biological Sciences, located at the State University of Londrina (UEL, Londrina, Brazil). Sowing was carried out in plastic pots with a capacity of 1.16 L, measuring 10.5 cm in height and 14.5 cm in upper diameter. Each pot was filled with approximately 0.750 kg of Carolina Soil^®^, class LXXVI 75H, previously moistened and homogenized. This substrate consisted of expanded vermiculite, sphagnum peat, dolomitic limestone, NPK fertilizer, and agricultural gypsum. Three seeds were sown per pot at a depth of approximately 2 cm. At 12 days after sowing (DAS), a single seedling was retained per pot following thinning. Each pot containing one plant was considered one experimental unit.

During the initial stage of the experiment, soybean plants were grown in a greenhouse under reduced light (50% of sunlight retention), achieved using a shade net. At 24 DAS, the plants were subjected to the assigned treatments: deionized water used as a negative control, a commercial carbon dots-based product (Arbolina^®^) applied at 0.20 mg mL^−1^ as a reference, and three concentrations of the Cdot formulation (0.02, 0.05, and 0.20 mg mL^−1^), diluted in deionized water on the day of application. These concentrations were defined based on prior screening assays. The 0.05 mg mL^−1^ concentration corresponded to the optimal biological response identified in preliminary trials, whereas 0.20 mg mL^−1^ matched the commercial reference dose. A tenfold dilution of this concentration (0.02 mg mL^−1^) was included for comparison. Each treatment included eight replicates. Foliar applications were performed using a garden hand compression manual backpack pressure pump sprayer (Palisad^®^), applying 100 mL of the respective solution across all eight replicates simultaneously. After application, at the end of the day, the plants were transferred to a greenhouse without shading (no shade net), where light attenuation was due only to the 0.15 mm-thick plastic covering, resulting in higher light intensities to promote light stress. The plants were maintained under these conditions for 14 days for subsequent physiological and biometric evaluations. The greenhouses had no active control of light or temperature, so plants were exposed to natural environmental fluctuations, which were monitored throughout the experimental period.

### 4.3. Physiological Evaluations

**Figure 10:**
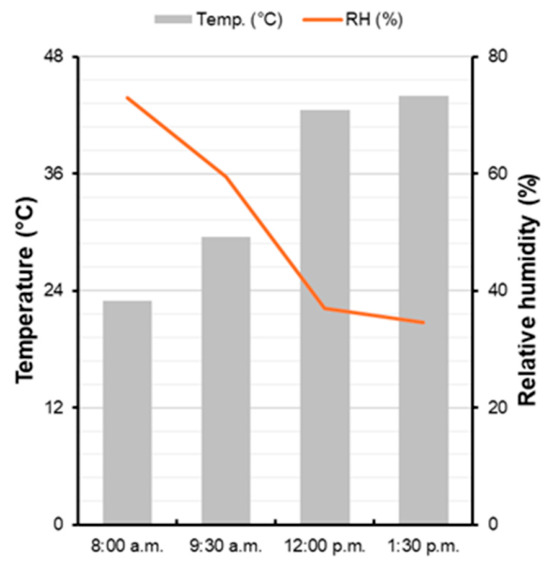
Average air temperature and relative humidity recorded during morning (08:00–9:30 a.m.) and noon (12:00–1:30 p.m.) measurement periods in the greenhouse.

Chlorophyll *a* fluorescence parameters were determined using a portable OS1p fluorometer (Opti-Sciences, Hudson, NY, USA) on intact leaflets. Measurements were conducted on the second trifoliate leaf, fully expanded, under shaded conditions, on the first and eighth days after transferring the pots to the high-light greenhouse. On the eighth day after transfer to the high-light environment, the measurements were carried out on the fourth trifoliate leaf, fully expanded, under high-light conditions. Before measurements, the leaves were dark-adapted for 15 min using FL-DC clips. Basal fluorescence (F_0_) was recorded using a weak modulated light applied for 0.1 s at 10% intensity. The leaves were then exposed to a saturating light pulse of 8250 µmol m^−2^ s^−1^ for 0.8 s to determine the maximum fluorescence (F_m_), variable fluorescence (F_v_), and maximum quantum yield of PS II (F_v_/F_m_) [43]. Measurements were performed at 08:00 a.m. and at noon. On the evaluation days, the mean photosynthetically active radiation (PAR) was approximately 350 µmol m^−2^ s^−1^ in the morning and 1100 µmol m^−2^ s^−1^ at noon. The average temperature and relative humidity during the morning and noon measurements are shown in Figure 10.

Temperature and relative humidity in the greenhouse were monitored during the experimental period using EL-USB-2 RH/TEMP data loggers (Lascar electronics, Erie, PA, USA).

DP(%) was calculated as an operational descriptor of the midday decrease in PSII efficiency, without implying a specific underlying mechanism (1):

(1) $$DP\left(\%\right)=\frac{F_{v}/F_{m\;\left(morning\right)-\;}F_{v}/F_{m\;(noon)}}{F_{v}/F_{m\;(morning)}}\;\times \;100$$

Gas exchange measurements were conducted in the morning, between 8:00 and 11:00 h, using a portable infrared gas analyzer (IRGA), LI-6400 XT (LI-COR^©^ Biosciences, Lincoln, NE, USA), equipped with a 6 cm^2^ leaf chamber. During measurements, the photosynthetic photon flux density was maintained at a saturating level of 1500 μmol m^−2^ s^−1^. Evaluations were performed on the second trifoliate leaf on the second day, and on the fourth trifoliate leaf on the 14th day after transferring the plants to the high-light environment. The net photosynthetic rate (*A*), stomatal conductance (*g*_s_), transpiration rate (*E*), and intercellular CO_2_ concentration (*C*_i_) were determined. Based on these parameters, intrinsic water use efficiency (iWUE) was calculated as the ratio *A*/*g*_s_, and instantaneous carboxylation efficiency (*k*) was estimated as the ratio *A*/*C*_i_.

At 36 DAS, the chlorophyll content index (CCI) was measured using the portable chlorophyll content meter atLEAF^®^ CHL PLUS (FT Green LLC, Washington, DC, USA), standardized on the fourth trifoliate leaf (*n* = 8). Measurements were taken at two points on each side of the three leaflets, resulting in six readings per leaf. For each treatment replicate, the CCI value was calculated as the mean of six measurements.

### 4.4. Biometric Evaluations

At 38 DAS, the experiment was dismantled. Shoot length (SL) was measured using a scale (cm), from the plant base to the apical meristem. For leaf area (LA) determination, all leaves were collected, and LA was immediately measured using a portable leaf area meter LI-3000C (LI-COR^©^ Biosciences, Lincoln, NE, USA). Subsequently, the leaves were oven-dried at 60 °C until constant weight to determine leaf dry mass (LDM).

Plants were removed from the pots, and roots were washed over a fine-mesh sieve under running water until complete removal of the substrate. Root length (RL) was measured as the primary taproot length, from the base of the stem to the root tip, using a scale (cm). Thereafter, roots and stems were placed in labeled paper bags and oven-dried at 60 °C, model SP-100 (SP Labor, Presidente Prudente, SP, BR) until constant weight to determine root dry mass (RDM) and stem dry mass (SDM). All dry masses were determined using a semi-analytical balance, model S203H (Bel Engineering, Monza, ITA). Total dry mass per plant (TDM) was obtained by summing leaf, stem, and root dry masses.

Relative growth rate (RGR) of the shoot and roots, as well as net assimilation rate (NAR), were calculated according to Magalhães [45]. Initial dry masses (Wi), as well as initial leaf area (LAi), were obtained from the destructive sampling of ten additional plants at the start of the treatments, following the same methodology. These values were used to calculate RGR and NAR. After 14 days of treatment, the experiment was dismantled and final dry masses (Wf) and final leaf area (LAf) were determined. RGR and NAR were calculated using Equations (2) and (3), respectively:

(2) $$RGR=\frac{\left(lnln\;Wf\;-lnln\;Wi\;\right)}{14}$$

(3) $$NAR=\frac{\left[\left(Wf-Wi\right)\times \left(lnln\;LAf-lnln\;LAi\;\right)\right]}{\left[\left(LAf-LAi\right)\times \left(14\right)\right]}$$

For the calculation of RGR of the shoot or roots, Wi and Wf correspond to the initial and final dry masses, respectively. For the calculation of NAR, Wi and Wf correspond to the initial and final total dry mass per plant, respectively.

### 4.5. Statistical Analysis

The assumptions of analysis of variance (ANOVA) were assessed using the Shapiro–Wilk test for normality and the Bartlett test for homogeneity of variances. Data were then subjected to ANOVA using the F-test, and when significant, means were compared using Tukey’s test at *p* < 0.05 or *p* < 0.10 (marginally significant), as specified in the figure captions. All statistical analyses were performed using R statistical software v. 4.5.1 and RStudio statistical software v. 2025.09.2+418 [46].

## 5. Conclusions

The results of the current study demonstrate a dose-dependent effect of Cdots on soybean physiology. The highest concentration (0.20 mg mL^−1^) increased dynamic photoinhibition, negatively affecting leaf expansion. In contrast, the lowest dose (0.02 mg mL^−1^) led to moderate improvements, although the responses were less consistent. The intermediate concentration (0.05 mg mL^−1^) stood out as the most effective, being associated with greater PSII stability during the initial acclimation to high irradiance (alleviation of chronic photoinhibition), higher chlorophyll levels, and greater biomass accumulation. Overall, these results suggest that Cdots can act as nanobiostimulants, improving plant performance under high-light conditions.

## Figures and Tables

**Table 1:** Shoot length (SL), root length (RL), leaf area (LA), leaf dry mass (LDM), stem dry mass (SDM), root dry mass (RDM), and total dry mass (TDM) of soybean plants (*Glycine max* L. Merr. cv. BRS 1054 IPRO) treated with a carbon dot (Cdot) formulation at three different concentrations (0.02, 0.05, and 0.20 mg mL^−1^), as well as with a carbon dot-based commercial product applied at the manufacturer-recommended dose (0.20 mg mL^−1^).

	SL		RL		LA		LDM		SDM		RDM		TDM	
	(cm)				(cm^2^)		(g)							
Commercial	52.9	b	21.4	a	290.44	ab	0.82	ab	0.90	ab	0.58	ab	2.3	abc
Control	53.3	b	19.1	a	297.36	ab	0.76	b	0.74	b	0.49	b	1.9	c
Cdot 0.02	60.3	ab	24.5	a	326.45	ab	0.89	ab	1.05	a	0.55	ab	2.4	ab
Cdot 0.05	66.7	a	22.0	a	337.53	a	0.94	a	1.06	a	0.63	a	2.6	a
Cdot 0.20	54.2	b	21.6	a	275.24	b	0.79	ab	0.87	ab	0.52	ab	2.1	bc
CV (%)	13.39		19.63		12.52		16.78		14.76		15.66		10.38	

Results are expressed as means (*n* = 8). Means followed by the same letter within a column do not differ significantly according to Tukey’s test (*p* < 0.05; LDM *p* < 0.10).

## Data Availability

The raw data supporting the conclusions of this article are included as Supplementary Materials.

## Supplementary Materials

The following supporting information can be downloaded at: https://www.mdpi.com/article/10.3390/plants15101446/s1.

## Abbreviations

The following abbreviations are used in this manuscript:

PSI	Photosystem I
PSII	Photosystem II
Cdots	Carbon dots
F_v_/F_m_	Maximum quantum yield of PSII
F_0_	Basal fluorescence
F_m_	Maximum fluorescence
F_v_	Variable fluorescence
DP (%)	Dynamic photoinhibition of PSII
PAR	Photosynthetically active radiation
IRGA	Infrared gas analyzer
*A*	Net photosynthetic rate
*g*_s_	Stomatal conductance
*E*	Transpiration rate
*C*_i_	Intercellular CO_2_ concentration
iWUE	Intrinsic water use efficiency
*k*	Instantaneous carboxylation efficiency
CCI	Chlorophyll content index
SL	Shoot length
RL	Root length
LA	Leaf area
LDM	Leaf dry mass
STM	Stem dry mass
RDM	Root dry mass
TDM	Total dry mass per plant
RGR	Relative growth rate
NAR	Net assimilation rate
Wi	Initial dry mass
LAi	Initial leaf area
Wf	Final dry mass
LAf	Final leaf area
ANOVA	Analysis of variance
p	p-valor
CV (%)	Coefficient of variation
PL	Spectrofluorimetric analysis
λₑₓ	Excitation wavelength
*Q*_a_	Quinone A
ROS	Reactive oxygen species
O_2_^•−^	Superoxide radicals
H_2_O_2_	Hydrogen peroxide
^•^OH	Hydroxyl radicals
^1^O_2_	Singlet oxygen
QY	Quantum yield
UV-Vis	Ultraviolet–visible spectroscopy
H_2_SO_4_	Sulfuric acid
TEM	Transmission electron microscopy
EMBRAPA	Brazilian Agricultural Research Corporation
UEL	University of Londrina
UFMT	Federal University of Mato Grosso
DAS	Days after sowing
CNPq	National Council for Scientific and Technological Development
FAPEMAT	Research Support Foundation of the State of Mato Grosso
MCTI	Ministry of Science, Technology and Innovation
INCT	National Institutes of Science and Technology
