# Supplementary material for: Applying Carbon Dots to Alleviate Photoinhibition and Boost Early Growth of Soybean Plants
Source: Plants (Basel). 2026 May 9;15(10):1446. doi: 10.3390/plants15101446 (PMC13210945; doi:10.3390/plants15101446)
Supplement: Supplementary file 1 [file plants-15-01446-s001.zip › plants-4196199-supplementary.pdf]

Supplementary Materials

Supplementary Methods: Statistical Analysis (ANOVA)

Figure 3

F0_M (morning)

Analysis of Variance Table

	DF	SS	MS	Fc	Pr>Fc
Treatament	4	382.7	95.69	0.29756	0.87755
Residuals	35	11255.3	321.58		
Total	39	11638.0			

Shapiro-Wilk normality test
p-value: 0.04451331
WARNING: at 5% of significance, residuals can not be considered normal!

Homogeneity of variances test
p-value: 0.5890488
According to the test of bartlett at 5% of significance, residuals can be considered homocedastic.

According to the F test, the means can not be considered distinct.

	Levels	Means
1	Cdot_0.020	173.750
2	Cdot_0.050	176.125
3	Cdot_0.20	180.500
4	Commercial	176.000
5	Control	171.125

F0_N (Noon)

Analysis of Variance Table

	DF	SS	MS	Fc	Pr>Fc
Treatament	4	30522	7630.6	2.5841	0.053887
Residuals	35	103353	2953.0		
Total	39	133876			

Shapiro-Wilk normality test
p-value: 0.2045864
According to Shapiro-Wilk normality test at 5% of significance, residuals can be considered normal.

Homogeneity of variances test
p-value: 0.6851797
According to the test of bartlett at 5% of significance, residuals can be considered homocedastic.

Tukey's test

Groups	Treatments	Means
a	Cdot_0.20	298
ab	Commercial	274.25
ab	Control	239.125
ab	Cdot_0.020	235.75
b	Cdot_0.050	223.75

Fv/Fm_M

Analysis of Variance Table

	DF	SS	MS	Fc	Pr>Fc
Treatament	4	0.058264	0.0145661	3.249	0.022851
Residuals	35	0.156914	0.0044833		
Total	39	0.215178			

Shapiro-Wilk normality test
p-value: 0.5303843
According to Shapiro-Wilk normality test at 5% of significance, residuals can be considered normal.

Homogeneity of variances test
p-value: 0.1982953
According to the test of bartlett at 5% of significance, residuals can be considered homocedastic.

Tukey's test

Groups	Treatments	Means
a	Cdot_0.050	0.6775125
a	Commercial	0.67675
ab	Cdot_0.020	0.650625
ab	Cdot_0.20	0.624
b	Control	0.5755

Fv/Fm_N

Analysis of Variance Table

	DF	SS	MS	Fc	Pr>Fc
Treatament	4	0.50683	0.126708	4.3096	0.0061276
Residuals	35	1.02906	0.029402		
Total	39	1.53589			

Shapiro-Wilk normality test
p-value: 0.2153926
According to Shapiro-Wilk normality test at 5% of significance, residuals can be considered normal.

Homogeneity of variances test
p-value: 0.8483642
According to the test of bartlett at 5% of significance, residuals can be considered homocedastic.

Tukey's test

Groups	Treatments	Means
a	Control	0.46025
a	Cdot_0.050	0.43375
ab	Cdot_0.020	0.393125
ab	Commercial	0.2435
b	Cdot_0.20	0.17275

DP(%)

Analysis of Variance Table

	DF	SS	MS	Fc	Pr>Fc
Treatament	4	12624	3155.9	6.055	0.00082139
Residuals	35	18242	521.2		
Total	39	30866			

Shapiro-Wilk normality test
p-value: 0.264537
According to Shapiro-Wilk normality test at 5% of significance, residuals can be considered normal.

Homogeneity of variances test
p-value: 0.8605329
According to the test of bartlett at 5% of significance, residuals can be considered homocedastic.

Tukey's test

Groups	Treatments	Means
a	Cdot_0.20	71.85224

```
ab      Commercial   60.92252
 bc     Cdot_0.050   36.87601
 bc     Cdot_0.020   36.25808
  c     Control      23.44241
------------------------------------------------------------------------
==========================================================================================
                                        Figure  4
==========================================================================================
                                          gs_M
------------------------------------------------------------------------


------------------------------------------------------------------------
Analysis of Variance Table
------------------------------------------------------------------------
           DF       SS        MS     Fc   Pr>Fc
Treatament  4 0.007506 0.0018764 1.8398 0.14322
Residuals  35 0.035695 0.0010199
Total      39 0.043201
------------------------------------------------------------------------
Shapiro-Wilk normality test
p-value:  0.08550382
According to Shapiro-Wilk normality test at 5% of significance, residuals can be
considered normal.
------------------------------------------------------------------------
Homogeneity of variances test
p-value:  0.5645312
According to the test of bartlett at 5% of significance, residuals can be considered
homocedastic.
------------------------------------------------------------------------
According to the F test, the means can not be considered distinct.
------------------------------------------------------------------------
      Levels     Means
1 Cdot_0.020 0.2065339
2 Cdot_0.050 0.1991848
3  Cdot_0.20 0.1895198
4 Commercial 0.2129653
5    Control 0.1739056
------------------------------------------------------------------------
                                          gs_N
------------------------------------------------------------------------
Analysis of Variance Table
------------------------------------------------------------------------
           DF       SS         MS     Fc  Pr>Fc
Treatament  4 0.007527 0.00188165 2.5939 0.0532
Residuals  35 0.025390 0.00072542
Total      39 0.032916
------------------------------------------------------------------------
Shapiro-Wilk normality test
p-value:  0.8021388
According to Shapiro-Wilk normality test at 5% of significance, residuals can be
considered normal.
------------------------------------------------------------------------
Homogeneity of variances test
p-value:  0.8790252
According to the test of bartlett at 5% of significance, residuals can be considered
homocedastic.
------------------------------------------------------------------------
Tukey's test
------------------------------------------------------------------------
Groups Treatments Means
a       Control      0.1849846
ab      Cdot_0.20    0.179608
ab      Cdot_0.020   0.1640926
ab      Cdot_0.050   0.1537111
 b      Commercial   0.1504378
------------------------------------------------------------------------
                                          A_M
------------------------------------------------------------------------
------------------------------------------------------------------------
Analysis of Variance Table
```

	DF	SS	MS	Fc	Pr>Fc
Treatament	4	1.6376	0.40939	0.51312	0.72647
Residuals	35	27.9247	0.79785		
Total	39	29.5622			

Shapiro-Wilk normality test
p-value: 0.2056827
According to Shapiro-Wilk normality test at 5% of significance, residuals can be considered normal.

Homogeneity of variances test
p-value: 0.7663441
According to the test of bartlett at 5% of significance, residuals can be considered homocedastic.

According to the F test, the means can not be considered distinct.

	Levels	Means
1	Cdot_0.020	8.403096
2	Cdot_0.050	8.437691
3	Cdot_0.20	8.156814
4	Commercial	8.690943
5	Control	8.145012

A_N

Analysis of Variance Table

	DF	SS	MS	Fc	Pr>Fc
Treatament	4	11.524	2.8809	1.1525	0.34836
Residuals	35	87.491	2.4997		
Total	39	99.014			

Shapiro-Wilk normality test
p-value: 0.7366641
According to Shapiro-Wilk normality test at 5% of significance, residuals can be considered normal.

Homogeneity of variances test
p-value: 0.1172934
According to the test of bartlett at 5% of significance, residuals can be considered homocedastic.

According to the F test, the means can not be considered distinct.

	Levels	Means
1	Cdot_0.020	5.883296
2	Cdot_0.050	6.508383
3	Cdot_0.20	6.485602
4	Commercial	5.099633
5	Control	5.603667

E_M

Analysis of Variance Table

	DF	SS	MS	Fc	Pr>Fc
Treatament	4	0.3162	0.079055	0.28539	0.88547
Residuals	35	9.6953	0.277010		
Total	39	10.0116			

Shapiro-Wilk normality test
p-value: 0.7792157
According to Shapiro-Wilk normality test at 5% of significance, residuals can be considered normal.

Homogeneity of variances test
p-value: 0.2079941
According to the test of bartlett at 5% of significance, residuals can be considered homocedastic.

```
------------------------------------------------------------------------
According to the F test, the means can not be considered distinct.
------------------------------------------------------------------------
      Levels    Means
1 Cdot_0.020 4.308508
2 Cdot_0.050 4.193948
3  Cdot_0.20 4.100757
4 Commercial 4.207920
5    Control 4.053468
------------------------------------------------------------------------
                                           E_N
------------------------------------------------------------------------
Analysis of Variance Table
------------------------------------------------------------------------
           DF     SS     MS     Fc  Pr>Fc
Treatament  4 10.560 2.6399 1.4778 0.2299
Residuals  35 62.523 1.7864
Total      39 73.083
------------------------------------------------------------------------
Shapiro-Wilk normality test
p-value:  0.2763478
According to Shapiro-Wilk normality test at 5% of significance, residuals can be
considered normal.
------------------------------------------------------------------------
Homogeneity of variances test
p-value:  0.9661429
According to the test of bartlett at 5% of significance, residuals can be considered
homocedastic.
------------------------------------------------------------------------
According to the F test, the means can not be considered distinct.
------------------------------------------------------------------------
      Levels    Means
1 Cdot_0.020 8.554985
2 Cdot_0.050 8.613348
3  Cdot_0.20 8.689571
4 Commercial 7.802297
5    Control 9.421430
------------------------------------------------------------------------
                                          Ci_M
------------------------------------------------------------------------
Analysis of Variance Table
------------------------------------------------------------------------
           DF      SS     MS    Fc    Pr>Fc
Treatament  4  385.17 96.293 2.273 0.080996
Residuals  35 1482.74 42.364
Total      39 1867.91
------------------------------------------------------------------------
Shapiro-Wilk normality test
p-value:  0.9209133
According to Shapiro-Wilk normality test at 5% of significance, residuals can be
considered normal.
------------------------------------------------------------------------
Homogeneity of variances test
p-value:  0.09933348
According to the test of bartlett at 5% of significance, residuals can be considered
homocedastic.
------------------------------------------------------------------------
Tukey's test
------------------------------------------------------------------------
Groups Treatments Means
a      Commercial   324.5685
ab     Cdot_0.050   322.2647
ab     Cdot_0.020   321.3999
ab     Control      317.7002
 b     Cdot_0.20    316.0406
------------------------------------------------------------------------
                                          Ci_N
------------------------------------------------------------------------
Analysis of Variance Table
------------------------------------------------------------------------
           DF     SS      MS     Fc   Pr>Fc
```

```
Treatament  4  691.2 172.795 1.7845 0.15403
Residuals  35 3389.2  96.834
Total      39 4080.4
------------------------------------------------------------------------
Shapiro-Wilk normality test
p-value:  0.8136541
According to Shapiro-Wilk normality test at 5% of significance, residuals can be
considered normal.
------------------------------------------------------------------------
Homogeneity of variances test
p-value:  0.5820476
According to the test of bartlett at 5% of significance, residuals can be considered
homocedastic.
------------------------------------------------------------------------
According to the F test, the means can not be considered distinct.
------------------------------------------------------------------------
      Levels    Means
1 Cdot_0.020 293.5383
2 Cdot_0.050 290.0312
3  Cdot_0.20 301.2354
4 Commercial 298.3686
5    Control 299.6440
------------------------------------------------------------------------
```

iWUE_M

```
------------------------------------------------------------------------
Analysis of Variance Table
------------------------------------------------------------------------
           DF     SS     MS     Fc   Pr>Fc
Treatament  4 102.08 25.519 1.7071 0.17049
Residuals  35 523.22 14.949
Total      39 625.30
------------------------------------------------------------------------
Shapiro-Wilk normality test
p-value:  0.3228807
According to Shapiro-Wilk normality test at 5% of significance, residuals can be
considered normal.
------------------------------------------------------------------------
Homogeneity of variances test
p-value:  0.7723835
According to the test of bartlett at 5% of significance, residuals can be considered
homocedastic.
------------------------------------------------------------------------
According to the F test, the means can not be considered distinct.
------------------------------------------------------------------------
      Levels    Means
1 Cdot_0.020 40.32426
2 Cdot_0.050 41.45411
3  Cdot_0.20 41.16652
4 Commercial 42.56152
5    Control 44.94695
------------------------------------------------------------------------
```

iWUE_N

```
------------------------------------------------------------------------
Analysis of Variance Table
------------------------------------------------------------------------
           DF      SS     MS      Fc   Pr>Fc
Treatament  4  186.04 46.509 0.96195 0.44053
Residuals  35 1692.22 48.349
Total      39 1878.26
------------------------------------------------------------------------
Shapiro-Wilk normality test
p-value:  0.3993942
According to Shapiro-Wilk normality test at 5% of significance, residuals can be
considered normal.
------------------------------------------------------------------------
Homogeneity of variances test
p-value:  0.78891
According to the test of bartlett at 5% of significance, residuals can be considered
homocedastic.
------------------------------------------------------------------------
According to the F test, the means can not be considered distinct.
```

```
------------------------------------------------------------------------
      Levels    Means
1 Cdot_0.020 39.26300
2 Cdot_0.050 41.76568
3  Cdot_0.20 35.34103
4 Commercial 37.19048
5    Control 37.71711
------------------------------------------------------------------------
```

k_M

```
------------------------------------------------------------------------
Analysis of Variance Table
------------------------------------------------------------------------
           DF        SS         MS      Fc   Pr>Fc
Treatement  4 0.0014876 0.00037190 0.45144 0.77063
Residuals  35 0.0288335 0.00082381
Total      39 0.0303211
------------------------------------------------------------------------
Shapiro-Wilk normality test
p-value:  0.1489431
According to Shapiro-Wilk normality test at 5% of significance, residuals can be
considered normal.
------------------------------------------------------------------------
Homogeneity of variances test
p-value:  0.7664136
According to the test of bartlett at 5% of significance, residuals can be considered
homocedastic.
------------------------------------------------------------------------
According to the F test, the means can not be considered distinct.
------------------------------------------------------------------------
      Levels     Means
1 Cdot_0.020 0.2797577
2 Cdot_0.050 0.2835567
3  Cdot_0.20 0.2753156
4 Commercial 0.2900600
5    Control 0.2728870
------------------------------------------------------------------------
```

k_N

```
------------------------------------------------------------------------
Analysis of Variance Table
------------------------------------------------------------------------
           DF       SS        MS      Fc   Pr>Fc
Treatement  4 0.015156 0.0037890 0.97028 0.43611
Residuals  35 0.136677 0.0039051
Total      39 0.151833
------------------------------------------------------------------------
Shapiro-Wilk normality test
p-value:  0.581577
According to Shapiro-Wilk normality test at 5% of significance, residuals can be
considered normal.
------------------------------------------------------------------------
Homogeneity of variances test
p-value:  0.1042814
According to the test of bartlett at 5% of significance, residuals can be considered
homocedastic.
------------------------------------------------------------------------
According to the F test, the means can not be considered distinct.
------------------------------------------------------------------------
      Levels     Means
1 Cdot_0.020 0.2150798
2 Cdot_0.050 0.2439063
3  Cdot_0.20 0.2335423
4 Commercial 0.1866137
5    Control 0.2208691
------------------------------------------------------------------------
========================================================================
```

Figure 5

```
========================================================================
```

F0_M

```
------------------------------------------------------------------------
Analysis of Variance Table
```

```
------------------------------------------------------------------------
           DF      SS     MS     Fc   Pr>Fc
Treatement  4  352.25 88.062 1.6703 0.17889
Residuals  35 1845.25 52.721
Total      39 2197.50
------------------------------------------------------------------------
Shapiro-Wilk normality test
p-value:  0.3121579
According to Shapiro-Wilk normality test at 5% of significance, residuals can be
considered normal.
------------------------------------------------------------------------
Homogeneity of variances test
p-value:  0.5886705
According to the test of bartlett at 5% of significance, residuals can be considered
homocedastic.
------------------------------------------------------------------------
According to the F test, the means can not be considered distinct.
------------------------------------------------------------------------
      Levels   Means
1 Cdot_0.020 152.250
2 Cdot_0.050 144.625
3  Cdot_0.20 146.875
4 Commercial 151.250
5    Control 146.250
------------------------------------------------------------------------
```

F0_N

```
------------------------------------------------------------------------
Analysis of Variance Table
------------------------------------------------------------------------
           DF      SS     MS     Fc    Pr>Fc
Treatement  4  2752.8 688.19 2.9863 0.031986
Residuals  35  8065.6 230.45
Total      39 10818.4
------------------------------------------------------------------------
Shapiro-Wilk normality test
p-value:  0.1732055
According to Shapiro-Wilk normality test at 5% of significance, residuals can be
considered normal.
------------------------------------------------------------------------
Homogeneity of variances test
p-value:  0.1839541
According to the test of bartlett at 5% of significance, residuals can be considered
homocedastic.
------------------------------------------------------------------------
Tukey's test
------------------------------------------------------------------------
Groups Treatments Means
a      Control      200.125
ab     Cdot_0.020   191.375
ab     Commercial   190.25
ab     Cdot_0.20    181.25
 b     Cdot_0.050   176.375
------------------------------------------------------------------------
```

Fv/Fm_M

```
------------------------------------------------------------------------
Analysis of Variance Table
------------------------------------------------------------------------
           DF        SS         MS      Fc   Pr>Fc
Treatement  4 0.0001563 3.9087e-05 0.14114 0.96572
Residuals  35 0.0096933 2.7695e-04
Total      39 0.0098496
------------------------------------------------------------------------
Shapiro-Wilk normality test
p-value:  0.4794896
According to Shapiro-Wilk normality test at 5% of significance, residuals can be
considered normal.
------------------------------------------------------------------------
Homogeneity of variances test
p-value:  0.1946604
According to the test of bartlett at 5% of significance, residuals can be considered
homocedastic.
```

```
-----------------------------------------------------------------------
According to the F test, the means can not be considered distinct.
-----------------------------------------------------------------------
      Levels    Means
1 Cdot_0.020 0.735125
2 Cdot_0.050 0.736625
3  Cdot_0.20 0.734250
4 Commercial 0.730750
5    Control 0.735250
-----------------------------------------------------------------------
```

Fv/Fm_N

```
-----------------------------------------------------------------------
Analysis of Variance Table
-----------------------------------------------------------------------
           DF       SS        MS     Fc   Pr>Fc
Treatament  4 0.026179 0.0065448 1.5687 0.20429
Residuals  35 0.146025 0.0041722
Total      39 0.172204
-----------------------------------------------------------------------
Shapiro-Wilk normality test
p-value:  0.1634663
According to Shapiro-Wilk normality test at 5% of significance, residuals can be
considered normal.
-----------------------------------------------------------------------
Homogeneity of variances test
p-value:  0.1998993
According to the test of bartlett at 5% of significance, residuals can be considered
homocedastic.
-----------------------------------------------------------------------
According to the F test, the means can not be considered distinct.
-----------------------------------------------------------------------
      Levels    Means
1 Cdot_0.020 0.601250
2 Cdot_0.050 0.647625
3  Cdot_0.20 0.632250
4 Commercial 0.588750
5    Control 0.581125
-----------------------------------------------------------------------
```

DP (%)

```
-----------------------------------------------------------------------
Analysis of Variance Table
-----------------------------------------------------------------------
           DF     SS     MS      Fc   Pr>Fc
Treatament  4  312.8 78.197 0.94241 0.45102
Residuals  35 2904.1 82.975
Total      39 3216.9
-----------------------------------------------------------------------
Shapiro-Wilk normality test
p-value:  0.2680898
According to Shapiro-Wilk normality test at 5% of significance, residuals can be
considered normal.
-----------------------------------------------------------------------
Homogeneity of variances test
p-value:  0.4837237
According to the test of bartlett at 5% of significance, residuals can be considered
homocedastic.
-----------------------------------------------------------------------
According to the F test, the means can not be considered distinct.
-----------------------------------------------------------------------
      Levels    Means
1 Cdot_0.020 21.40131
2 Cdot_0.050 14.11574
3  Cdot_0.20 16.07007
4 Commercial 19.92566
5    Control 20.39530
-----------------------------------------------------------------------
=========================================================================================
```

Figure 6

```
=========================================================================================
```

F0_M

```
-----------------------------------------------------------------------
```

```
Analysis of Variance Table
------------------------------------------------------------------------
           DF      SS     MS     Fc   Pr>Fc
Treatament  4  260.35 65.087 1.6512 0.18342
Residuals  35 1379.63 39.418
Total      39 1639.98
------------------------------------------------------------------------
Shapiro-Wilk normality test
p-value:  0.5710049
According to Shapiro-Wilk normality test at 5% of significance, residuals can be
considered normal.
------------------------------------------------------------------------
Homogeneity of variances test
p-value:  0.2047757
According to the test of bartlett at 5% of significance, residuals can be considered
homocedastic.
------------------------------------------------------------------------
According to the F test, the means can not be considered distinct.
------------------------------------------------------------------------
      Levels   Means
1 Cdot_0.020 128.375
2 Cdot_0.050 133.250
3  Cdot_0.20 136.125
4 Commercial 133.250
5    Control 131.375
------------------------------------------------------------------------
```

F0_N

```
------------------------------------------------------------------------
Analysis of Variance Table
------------------------------------------------------------------------
           DF      SS      MS     Fc    Pr>Fc
Treatament  4  6451.8 1612.94 2.9199 0.034844
Residuals  35 19333.6  552.39
Total      39 25785.4
------------------------------------------------------------------------
Shapiro-Wilk normality test
p-value:  0.9091635
According to Shapiro-Wilk normality test at 5% of significance, residuals can be
considered normal.
------------------------------------------------------------------------
Homogeneity of variances test
p-value:  0.3573561
According to the test of bartlett at 5% of significance, residuals can be considered
homocedastic.
------------------------------------------------------------------------
Tukey's test
------------------------------------------------------------------------
Groups Treatments Means
a       Cdot_0.050   226.5
ab      Cdot_0.20    219.125
ab      Cdot_0.020   218.875
 b      Commercial   196.375
 b      Control      196
------------------------------------------------------------------------
```

Fv/Fm_M

```
------------------------------------------------------------------------
Analysis of Variance Table
------------------------------------------------------------------------
           DF        SS         MS      Fc   Pr>Fc
Treatament  4 0.0005849 0.00014622 0.92316 0.46156
Residuals  35 0.0055439 0.00015840
Total      39 0.0061288
------------------------------------------------------------------------
Shapiro-Wilk normality test
p-value:  0.3016384
According to Shapiro-Wilk normality test at 5% of significance, residuals can be
considered normal.
------------------------------------------------------------------------
Homogeneity of variances test
p-value:  0.6270034
```

According to the test of bartlett at 5% of significance, residuals can be considered homocedastic.

According to the F test, the means can not be considered distinct.

	Levels	Means
1	Cdot_0.020	0.754000
2	Cdot_0.050	0.761750
3	Cdot_0.20	0.759000
4	Commercial	0.751125
5	Control	0.758750

Fv/Fm_N

Analysis of Variance Table

	DF	SS	MS	Fc	Pr>Fc
Treatament	4	0.05559	0.013898	1.312	0.28464
Residuals	35	0.37075	0.010593		
Total	39	0.42635			

Shapiro-Wilk normality test
p-value: 0.3782517
According to Shapiro-Wilk normality test at 5% of significance, residuals can be considered normal.

Homogeneity of variances test
p-value: 0.5829474
According to the test of bartlett at 5% of significance, residuals can be considered homocedastic.

According to the F test, the means can not be considered distinct.

	Levels	Means
1	Cdot_0.020	0.379625
2	Cdot_0.050	0.409750
3	Cdot_0.20	0.393875
4	Commercial	0.474375
5	Control	0.460500

DP (%)

Analysis of Variance Table

	DF	SS	MS	Fc	Pr>Fc
Treatament	4	1092.7	273.17	1.4399	0.24145
Residuals	35	6639.8	189.71		
Total	39	7732.4			

Shapiro-Wilk normality test
p-value: 0.2853159
According to Shapiro-Wilk normality test at 5% of significance, residuals can be considered normal.

Homogeneity of variances test
p-value: 0.5478745
According to the test of bartlett at 5% of significance, residuals can be considered homocedastic.

According to the F test, the means can not be considered distinct.

	Levels	Means
1	Cdot_0.020	49.52306
2	Cdot_0.050	46.28070
3	Cdot_0.20	47.23029
4	Commercial	36.56716
5	Control	37.96863

Figure 7

CCI

```
------------------------------------------------------------------------
Analysis of Variance Table
------------------------------------------------------------------------
           DF      SS      MS    Fc      Pr>Fc
Treatament  4  82.974 20.7436 7.257 0.00022949
Residuals  35 100.045  2.8584
Total      39 183.019
------------------------------------------------------------------------
Shapiro-Wilk normality test
p-value:  0.5457402
According to Shapiro-Wilk normality test at 5% of significance, residuals can be
considered normal.
------------------------------------------------------------------------
Homogeneity of variances test
p-value:  0.9596198
According to the test of bartlett at 5% of significance, residuals can be considered
homocedastic.
------------------------------------------------------------------------
Tukey's test
------------------------------------------------------------------------
Groups Treatments Means
a       Cdot_0.050   31.38333
ab      Cdot_0.20    29.43542
ab      Cdot_0.020   29.37708
 b      Commercial   27.7125
 b      Control      27.35208
------------------------------------------------------------------------
```

==

Figure 8

==

gs_M

```
------------------------------------------------------------------------
------------------------------------------------------------------------
Analysis of Variance Table
------------------------------------------------------------------------
           DF       SS        MS      Fc  Pr>Fc
Treatament  4 0.010089 0.0025223 0.79656 0.5387
Residuals  25 0.079162 0.0031665
Total      29 0.089251
------------------------------------------------------------------------
Shapiro-Wilk normality test
p-value:  0.9367457
According to Shapiro-Wilk normality test at 5% of significance, residuals can be
considered normal.
------------------------------------------------------------------------
Homogeneity of variances test
p-value:  0.7638018
According to the test of bartlett at 5% of significance, residuals can be considered
homocedastic.
------------------------------------------------------------------------
According to the F test, the means can not be considered distinct.
------------------------------------------------------------------------
      Levels     Means
1 Cdot_0.020 0.1637026
2 Cdot_0.050 0.1564261
3  Cdot_0.20 0.2038258
4 Commercial 0.1799201
5    Control 0.1971090
------------------------------------------------------------------------
```

gs_N

```
------------------------------------------------------------------------
Analysis of Variance Table
------------------------------------------------------------------------
           DF       SS        MS     Fc    Pr>Fc
Treatament  4 0.021811 0.0054527 2.3183 0.084917
Residuals  25 0.058801 0.0023520
Total      29 0.080612
------------------------------------------------------------------------
Shapiro-Wilk normality test
p-value:  0.9803
```

According to Shapiro-Wilk normality test at 5% of significance, residuals can be considered normal.

Homogeneity of variances test
p-value: 0.2764214
According to the test of bartlett at 5% of significance, residuals can be considered homocedastic.

Tukey's test

```
Groups Treatments Means
a       Cdot_0.20    0.1362127
ab      Cdot_0.020   0.1205924
ab      Control      0.1045238
ab      Commercial   0.09202741
 b      Cdot_0.050   0.05713149
```

A_M

Analysis of Variance Table

```
           DF      SS     MS     Fc   Pr>Fc
Treatament  4  26.824 6.7060 1.1045 0.37631
Residuals  25 151.782 6.0713
Total      29 178.605
```

Shapiro-Wilk normality test
p-value: 0.6365746
According to Shapiro-Wilk normality test at 5% of significance, residuals can be considered normal.

Homogeneity of variances test
p-value: 0.3007103
According to the test of bartlett at 5% of significance, residuals can be considered homocedastic.

According to the F test, the means can not be considered distinct.

```
      Levels     Means
1 Cdot_0.020  8.698784
2 Cdot_0.050  8.788266
3  Cdot_0.20 11.205191
4 Commercial 10.072059
5    Control  9.151112
```

A_N

Analysis of Variance Table

```
           DF     SS     MS     Fc   Pr>Fc
Treatament  4 102.48 25.621 2.0717 0.11479
Residuals  25 309.18 12.367
Total      29 411.66
```

Shapiro-Wilk normality test
p-value: 0.6127021
According to Shapiro-Wilk normality test at 5% of significance, residuals can be considered normal.

Homogeneity of variances test
p-value: 0.2913407
According to the test of bartlett at 5% of significance, residuals can be considered homocedastic.

According to the F test, the means can not be considered distinct.

```
      Levels    Means
1 Cdot_0.020 7.144741
2 Cdot_0.050 3.906627
3  Cdot_0.20 8.768858
4 Commercial 5.834350
```

```
5    Control 8.753048
------------------------------------------------------------------------
                                           E_M
------------------------------------------------------------------------
Analysis of Variance Table
------------------------------------------------------------------------
           DF      SS      MS     Fc   Pr>Fc
Treatament  4  2.7772 0.69429 1.0125 0.41984
Residuals  25 17.1425 0.68570
Total      29 19.9197
------------------------------------------------------------------------
Shapiro-Wilk normality test
p-value:  0.5440581
According to Shapiro-Wilk normality test at 5% of significance, residuals can be
considered normal.
------------------------------------------------------------------------
Homogeneity of variances test
p-value:  0.4081609
According to the test of bartlett at 5% of significance, residuals can be considered
homocedastic.
------------------------------------------------------------------------
According to the F test, the means can not be considered distinct.
------------------------------------------------------------------------
      Levels    Means
1 Cdot_0.020 2.311611
2 Cdot_0.050 2.602134
3  Cdot_0.20 3.196825
4 Commercial 2.855326
5    Control 2.960439

------------------------------------------------------------------------
                                           E_N
------------------------------------------------------------------------
Analysis of Variance Table
------------------------------------------------------------------------
           DF      SS      MS     Fc    Pr>Fc
Treatament  4  43.753 10.9383 2.1873 0.099621
Residuals  25 125.018  5.0007
Total      29 168.772
------------------------------------------------------------------------
Shapiro-Wilk normality test
p-value:  0.2173463
According to Shapiro-Wilk normality test at 5% of significance, residuals can be
considered normal.
------------------------------------------------------------------------
Homogeneity of variances test
p-value:  0.4938048
According to the test of bartlett at 5% of significance, residuals can be considered
homocedastic.
------------------------------------------------------------------------
Tukey's test
------------------------------------------------------------------------
Groups Treatments Means
a      Cdot_0.20    7.434029
ab     Cdot_0.020   7.117624
ab     Control      6.456422
ab     Commercial   6.010252
 b     Cdot_0.050   4.003251
------------------------------------------------------------------------
                                          Ci_M
------------------------------------------------------------------------
Analysis of Variance Table
------------------------------------------------------------------------
           DF     SS     MS      Fc   Pr>Fc
Treatament  4  786.9 196.73 0.71488 0.58964
Residuals  25 6879.9 275.20
Total      29 7666.9
------------------------------------------------------------------------
Shapiro-Wilk normality test
p-value:  0.3738691
```

```
According to Shapiro-Wilk normality test at 5% of significance, residuals can be
considered normal.
------------------------------------------------------------------------
Homogeneity of variances test
p-value:  0.09406023
According to the test of bartlett at 5% of significance, residuals can be considered
homocedastic.
------------------------------------------------------------------------
According to the F test, the means can not be considered distinct.
------------------------------------------------------------------------
      Levels    Means
1 Cdot_0.020 313.2950
2 Cdot_0.050 307.7243
3  Cdot_0.20 305.2203
4 Commercial 316.2369
5    Control 318.9039
------------------------------------------------------------------------
```

Ci_N

```
------------------------------------------------------------------------
Analysis of Variance Table
------------------------------------------------------------------------
           DF      SS     MS      Fc   Pr>Fc
Treatament  4  1162.4 290.60 0.56992 0.68685
Residuals  25 12747.2 509.89
Total      29 13909.6
------------------------------------------------------------------------
Shapiro-Wilk normality test
p-value:  0.2018963
According to Shapiro-Wilk normality test at 5% of significance, residuals can be
considered normal.
------------------------------------------------------------------------
Homogeneity of variances test
p-value:  0.755731
According to the test of bartlett at 5% of significance, residuals can be considered
homocedastic.
------------------------------------------------------------------------
According to the F test, the means can not be considered distinct.
------------------------------------------------------------------------
      Levels    Means
1 Cdot_0.020 256.4406
2 Cdot_0.050 250.2116
3  Cdot_0.20 246.4368
4 Commercial 251.5124
5    Control 237.8034
------------------------------------------------------------------------
```

iWUE_M

```
------------------------------------------------------------------------
Analysis of Variance Table
------------------------------------------------------------------------
           DF      SS     MS      Fc   Pr>Fc
Treatament  4  276.51 69.127 0.77658 0.55087
Residuals  25 2225.35 89.014
Total      29 2501.86
------------------------------------------------------------------------
Shapiro-Wilk normality test
p-value:  0.3362932
According to Shapiro-Wilk normality test at 5% of significance, residuals can be
considered normal.
------------------------------------------------------------------------
Homogeneity of variances test
p-value:  0.2260242
According to the test of bartlett at 5% of significance, residuals can be considered
homocedastic.
------------------------------------------------------------------------
According to the F test, the means can not be considered distinct.
------------------------------------------------------------------------
      Levels    Means
1 Cdot_0.020 55.01301
2 Cdot_0.050 57.37083
3  Cdot_0.20 57.36259
4 Commercial 51.23933
```

```
5    Control 50.12516
------------------------------------------------------------------------
                                                 iWUE_N
------------------------------------------------------------------------
Analysis of Variance Table
------------------------------------------------------------------------
           DF     SS     MS      Fc   Pr>Fc
Treatement  4  522.3 130.58 0.61456 0.65613
Residuals  25 5312.0 212.48
Total      29 5834.3
------------------------------------------------------------------------
Shapiro-Wilk normality test
p-value:  0.3071235
According to Shapiro-Wilk normality test at 5% of significance, residuals can be
considered normal.
------------------------------------------------------------------------
Homogeneity of variances test
p-value:  0.5835244
According to the test of bartlett at 5% of significance, residuals can be considered
homocedastic.
------------------------------------------------------------------------
According to the F test, the means can not be considered distinct.
------------------------------------------------------------------------
      Levels    Means
1 Cdot_0.020 57.92045
2 Cdot_0.050 62.21173
3  Cdot_0.20 65.45877
4 Commercial 60.74663
5    Control 70.06406
------------------------------------------------------------------------
                                                   k_M
------------------------------------------------------------------------
Analysis of Variance Table
------------------------------------------------------------------------
           DF       SS        MS     Fc   Pr>Fc
Treatement  4 0.051039 0.0127597 1.3019 0.29632
Residuals  25 0.245013 0.0098005
Total      29 0.296052
------------------------------------------------------------------------
Shapiro-Wilk normality test
p-value:  0.338616
According to Shapiro-Wilk normality test at 5% of significance, residuals can be
considered normal.
------------------------------------------------------------------------
Homogeneity of variances test
p-value:  0.2425474
According to the test of bartlett at 5% of significance, residuals can be considered
homocedastic.
------------------------------------------------------------------------
According to the F test, the means can not be considered distinct.
------------------------------------------------------------------------
      Levels     Means
1 Cdot_0.020 0.2976152
2 Cdot_0.050 0.3061547
3  Cdot_0.20 0.3983130
4 Commercial 0.3765483
5    Control 0.3784793
------------------------------------------------------------------------
                                                   k_N
------------------------------------------------------------------------
Analysis of Variance Table
------------------------------------------------------------------------
           DF      SS       MS     Fc   Pr>Fc
Treatement  4 0.17834 0.044586 1.5169 0.22756
Residuals  25 0.73480 0.029392
Total      29 0.91315
------------------------------------------------------------------------
Shapiro-Wilk normality test
p-value:  0.3323736
According to Shapiro-Wilk normality test at 5% of significance, residuals can be
considered normal.
```

```
------------------------------------------------------------------------
Homogeneity of variances test
p-value:  0.3753498
According to the test of bartlett at 5% of significance, residuals can be considered
homocedastic.
------------------------------------------------------------------------
According to the F test, the means can not be considered distinct.
------------------------------------------------------------------------
      Levels     Means
1 Cdot_0.020 0.3157641
2 Cdot_0.050 0.1529597
3  Cdot_0.20 0.3906251
4 Commercial 0.2792400
5    Control 0.2985033
------------------------------------------------------------------------
```

Figure 9

NAR

```
------------------------------------------------------------------------
Analysis of Variance Table
------------------------------------------------------------------------
           DF      SS        MS     Fc    Pr>Fc
Treatament  4 0.11833 0.0295832 3.3203 0.020871
Residuals  35 0.31184 0.0089098
Total      39 0.43018
------------------------------------------------------------------------
Shapiro-Wilk normality test
p-value:  0.9023702
According to Shapiro-Wilk normality test at 5% of significance, residuals can be
considered normal.
------------------------------------------------------------------------
Homogeneity of variances test
p-value:  0.4058372
According to the test of bartlett at 5% of significance, residuals can be considered
homocedastic.
------------------------------------------------------------------------
Tukey's test
------------------------------------------------------------------------
Groups Treatments Means
a      Cdot_0.050   0.6458548
ab     Cdot_0.020   0.6248444
ab     Commercial   0.5551068
ab     Cdot_0.20    0.534834
 b     Control      0.5021475
------------------------------------------------------------------------
```

RGR_S

```
------------------------------------------------------------------------
Analysis of Variance Table
------------------------------------------------------------------------
           DF     SS     MS    Fc     Pr>Fc
Treatament  4 2467.3 616.81 5.743 0.0011598
Residuals  35 3759.1 107.40
Total      39 6226.3
------------------------------------------------------------------------
Shapiro-Wilk normality test
p-value:  0.6260762
According to Shapiro-Wilk normality test at 5% of significance, residuals can be
considered normal.
------------------------------------------------------------------------
Homogeneity of variances test
p-value:  0.3790997
According to the test of bartlett at 5% of significance, residuals can be considered
homocedastic.
------------------------------------------------------------------------
Tukey's test
------------------------------------------------------------------------
Groups Treatments Means
a      Cdot_0.050   116.221
ab     Cdot_0.020   114.7888
abc    Commercial   105.0022
```

```
 bc     Cdot_0.20    100.0181
  c     Control      96.45276
------------------------------------------------------------------------
```

RGR_R

```
------------------------------------------------------------------------
Analysis of Variance Table
------------------------------------------------------------------------
           DF     SS     MS     Fc    Pr>Fc
Treatament  4 1853.3 463.34 3.8578 0.010643
Residuals  35 4203.6 120.10
Total      39 6057.0
------------------------------------------------------------------------
Shapiro-Wilk normality test
p-value:  0.3040365
According to Shapiro-Wilk normality test at 5% of significance, residuals can be
considered normal.
------------------------------------------------------------------------
Homogeneity of variances test
p-value:  0.4374286
According to the test of bartlett at 5% of significance, residuals can be considered
homocedastic.
------------------------------------------------------------------------
Tukey's test
------------------------------------------------------------------------
Groups Treatments Means
a       Cdot_0.050   150.9028
a       Cdot_0.020   149.2275
ab      Commercial   145.0035
ab      Cdot_0.20    137.4451
 b      Control      133.2179
------------------------------------------------------------------------
=========================================================================================
```

Table 1

```
=========================================================================================
```

SL

```
------------------------------------------------------------------------
Analysis of Variance Table
------------------------------------------------------------------------
           DF     SS      MS     Fc     Pr>Fc
Treatament  4 1152.5 288.117 4.8667 0.0031585
Residuals  35 2072.0  59.201
Total      39 3224.5
------------------------------------------------------------------------
Shapiro-Wilk normality test
p-value:  0.07679964
According to Shapiro-Wilk normality test at 5% of significance, residuals can be
considered normal.
------------------------------------------------------------------------
Homogeneity of variances test
p-value:  0.5052267
According to the test of bartlett at 5% of significance, residuals can be considered
homocedastic.
------------------------------------------------------------------------
Tukey's test
------------------------------------------------------------------------
Groups Treatments Means
a       Cdot_0.050   66.7375
ab      Cdot_0.020   60.3125
 b      Cdot_0.20    54.1625
 b      Control      53.25
 b      Commercial   52.85
------------------------------------------------------------------------
```

RL

```
------------------------------------------------------------------------
Analysis of Variance Table
------------------------------------------------------------------------
           DF    SS     MS     Fc  Pr>Fc
Treatament  4 117.4 29.349 1.6167 0.1919
Residuals  35 635.4 18.154
Total      39 752.8
------------------------------------------------------------------------
```

```
Shapiro-Wilk normality test
p-value:  0.2898883
According to Shapiro-Wilk normality test at 5% of significance, residuals can be
considered normal.
------------------------------------------------------------------------
Homogeneity of variances test
p-value:  0.07372724
According to the test of bartlett at 5% of significance, residuals can be considered
homocedastic.
------------------------------------------------------------------------
According to the F test, the means can not be considered distinct.
------------------------------------------------------------------------
      Levels   Means
1 Cdot_0.020 24.4500
2 Cdot_0.050 22.0125
3  Cdot_0.20 21.6000
4 Commercial 21.3625
5    Control 19.0750
------------------------------------------------------------------------
```

LA

```
------------------------------------------------------------------------
Analysis of Variance Table
------------------------------------------------------------------------
           DF    SS     MS    Fc    Pr>Fc
Treatament  4 21389 5347.2 3.655 0.013694
Residuals  35 51205 1463.0
Total      39 72594
------------------------------------------------------------------------
Shapiro-Wilk normality test
p-value:  0.6006749
According to Shapiro-Wilk normality test at 5% of significance, residuals can be
considered normal.
------------------------------------------------------------------------
Homogeneity of variances test
p-value:  0.5680418
According to the test of bartlett at 5% of significance, residuals can be considered
homocedastic.
------------------------------------------------------------------------
Tukey's test
------------------------------------------------------------------------
Groups Treatments Means
a      Cdot_0.050   337.5263
ab     Cdot_0.020   326.4513
ab     Control      297.3587
ab     Commercial   290.4412
 b     Cdot_0.20    275.235
------------------------------------------------------------------------
```

LDM

```
------------------------------------------------------------------------
Analysis of Variance Table
------------------------------------------------------------------------
           DF      SS       MS     Fc    Pr>Fc
Treatament  4 0.18092 0.045230 2.2686 0.081463
Residuals  35 0.69780 0.019937
Total      39 0.87872
------------------------------------------------------------------------
Shapiro-Wilk normality test
p-value:  0.8287186
According to Shapiro-Wilk normality test at 5% of significance, residuals can be
considered normal.
------------------------------------------------------------------------
Homogeneity of variances test
p-value:  0.7182087
According to the test of bartlett at 5% of significance, residuals can be considered
homocedastic.
------------------------------------------------------------------------
Tukey's test
------------------------------------------------------------------------
Groups Treatments Means
a      Cdot_0.050   0.943375
ab     Cdot_0.020   0.890875
```

```
ab      Commercial   0.824
ab      Cdot_0.20    0.793
 b      Control      0.757
------------------------------------------------------------------------
                                           SDM
------------------------------------------------------------------------
Analysis of Variance Table
------------------------------------------------------------------------
           DF      SS       MS     Fc      Pr>Fc
Treatament  4 0.55491 0.138727 7.4153 0.00019521
Residuals  35 0.65479 0.018708
Total      39 1.20970
------------------------------------------------------------------------
Shapiro-Wilk normality test
p-value:  0.7011448
According to Shapiro-Wilk normality test at 5% of significance, residuals can be
considered normal.
------------------------------------------------------------------------
Homogeneity of variances test
p-value:  0.0600515
According to the test of bartlett at 5% of significance, residuals can be considered
homocedastic.
------------------------------------------------------------------------
Tukey's test
------------------------------------------------------------------------
Groups Treatments Means
a       Cdot_0.050   1.060875
a       Cdot_0.020   1.0515
ab      Commercial   0.902
ab      Cdot_0.20    0.873
 b      Control      0.747
------------------------------------------------------------------------
                                           RDM
------------------------------------------------------------------------
Analysis of Variance Table
------------------------------------------------------------------------
           DF      SS         MS    Fc    Pr>Fc
Treatament  4 0.08349 0.0208725 2.782 0.041648
Residuals  35 0.26259 0.0075026
Total      39 0.34608
------------------------------------------------------------------------
Shapiro-Wilk normality test
p-value:  0.9167457
According to Shapiro-Wilk normality test at 5% of significance, residuals can be
considered normal.
------------------------------------------------------------------------
Homogeneity of variances test
p-value:  0.4515067
According to the test of bartlett at 5% of significance, residuals can be considered
homocedastic.
------------------------------------------------------------------------
Tukey's test
------------------------------------------------------------------------
Groups Treatments Means
a       Cdot_0.050   0.625125
ab      Commercial   0.577875
ab      Cdot_0.020   0.55
ab      Cdot_0.20    0.51775
 b      Control      0.495
------------------------------------------------------------------------
                                           TDM
------------------------------------------------------------------------
Analysis of Variance Table
------------------------------------------------------------------------
           DF     SS      MS     Fc      Pr>Fc
Treatament  4 1.9782 0.49455 8.5086 6.6228e-05
Residuals  35 2.0343 0.05812
Total      39 4.0125
------------------------------------------------------------------------
Shapiro-Wilk normality test
p-value:  0.7154804
```

```
According to Shapiro-Wilk normality test at 5% of significance, residuals can be
considered normal.
------------------------------------------------------------------------
Homogeneity of variances test
p-value:  0.204624
According to the test of bartlett at 5% of significance, residuals can be considered
homocedastic.
------------------------------------------------------------------------
Tukey's test
------------------------------------------------------------------------
Groups Treatments Means
a       Cdot_0.050   2.629375
ab      Cdot_0.020   2.492375
abc     Commercial   2.303875
 bc     Cdot_0.20    2.18375
  c     Control      1.999
------------------------------------------------------------------------
```
